# Non-ionotropic signaling through the NMDA receptor GluN2B carboxy-terminal domain drives dendritic spine plasticity and reverses fragile X phenotypes

**DOI:** 10.1016/j.celrep.2025.115311

**Published:** 2025-02-20

**Authors:** Stephanie A. Barnes, Aurore Thomazeau, Peter S.B. Finnie, Maxwell J. Heinrich, Arnold J. Heynen, Noburu H. Komiyama, Seth G.N. Grant, Frank S. Menniti, Emily K. Osterweil, Mark F. Bear

**Affiliations:** 1 The Picower Institute for Learning and Memory, Massachusetts Institute of Technology, Cambridge, MA 02139, USA; 2 Centre for Discovery Brain Sciences, University of Edinburgh, Hugh Robson Building, George Square, Edinburgh EH8 9XD, UK; 3 Centre for Clinical Brain Sciences, University of Edinburgh, Edinburgh EH16 4SB, UK; 4 Simons Initiative for the Developing Brain (SIDB), Centre for Discovery Brain Sciences, University of Edinburgh, Edinburgh EH8 9XD, UK; 5 The Patrick Wild Centre, Centre for Discovery Brain Sciences, University of Edinburgh, Edinburgh EH8 9XD, UK; 6 MindImmune Therapeutics, Inc., The George & Anne Ryan Institute for Neuroscience, University of Rhode Island, Kingston, RI 02881, USA; 7 F.M. Kirby Center for Neurobiology, Translational Neuroscience Center, Department of Neurology, Harvard Medical School, Boston Children’s Hospital, Boston, MA 02115, USA; 8 Lead contact

## Abstract

*N*-methyl-D-aspartate (NMDA)-induced spine shrinkage proceeds independently of ion flux and requires the initiation of *de novo* protein synthesis. Using subtype-selective pharmacological and genetic tools, we find that structural plasticity is dependent on ligand binding to GluN2B-containing NMDA receptors (NMDARs) and signaling via the GluN2B carboxy-terminal domain (CTD). Disruption of non-ionotropic signaling by replacing the GluN2B CTD with the GluN2A CTD leads to an increase in spine density, dysregulated basal protein synthesis, exaggerated long-term depression mediated by G-protein-coupled metabotropic glutamate receptors (mGluR-LTD), and epileptiform activity reminiscent of phenotypes observed in the *Fmr1* knockout (KO) model of fragile X syndrome. By crossing the *Fmr1* KO mice with animals in which the GluN2A CTD has been replaced with the GluN2B CTD, we observe a correction of these core fragile X phenotypes. These findings suggest that non-ionotropic NMDAR signaling through GluN2B may represent a novel therapeutic target for the treatment of fragile X and related causes of intellectual disability and autism.

## In brief

Barnes et al. used an assay of spine plasticity in hippocampal slices to study non-ionotropic NMDA-receptor signaling. Signaling is initiated via the GluN2B carboxy-terminal domain and modulates spine morphology, protein synthesis, and protein-synthesis-dependent synaptic plasticity. Augmenting GluN2B signaling reverses core phenotypes in the mouse model of fragile X syndrome.

## Graphical abstract

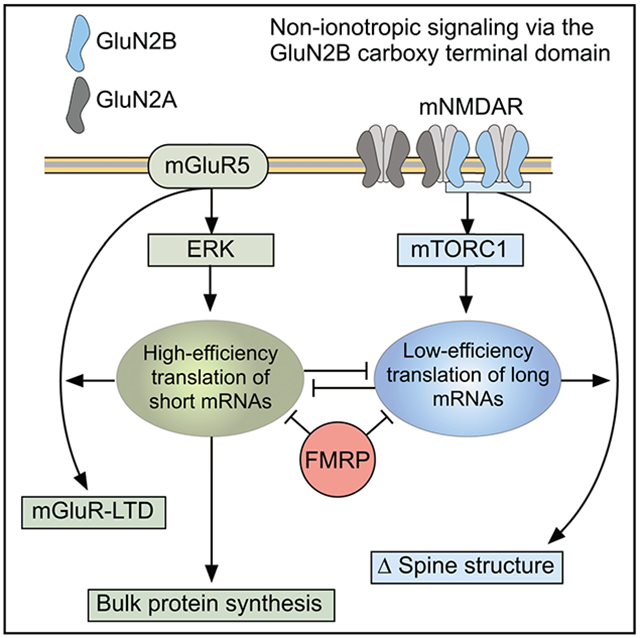

## INTRODUCTION

*N*-methyl-D-aspartate receptors (NMDARs) play a crucial role in refining neural circuits that are established during development and modified during experience-dependent learning. These excitatory glutamatergic receptors mediate dynamic changes in synaptic connectivity, which include the controlled regulation of dendritic spine formation and enlargement as well as spine retraction and elimination. Bidirectional changes in dendritic spine structure coincide with changes in synaptic strength. Enlargement of dendritic spines accompanies long-term potentiation (LTP), whereas spine shrinkage is observed after long-term depression (LTD). Considerable evidence suggests that perturbations in the maturation and plasticity of neuronal connections underlie neurological disorders associated with cognitive impairments. In agreement, numerous mutations in NMDAR subunits have been identified in patients with intellectual disability (ID), autism spectrum disorder (ASD), and epilepsy.^1–3^ These findings indicate that NMDAR dysfunction may play a prominent role in the pathophysiology of neurodevelopmental disorders (NDDs).

In the CA1 region of the hippocampus, NMDAR-dependent LTD and spine shrinkage can be induced by brief application of the agonist NMDA,^4–6^ low-frequency stimulation of identified input pathways,^7–9^ or uncaging glutamate at single spines.^10^ From these studies it has emerged that NMDARs can trigger intracellular events independently of ion permeation.^11–13^ Although there are conflicting data on the requirement for Ca^2+^ flux to trigger NMDAR-LTD,^10,14–23^ numerous groups have consistently shown that structural plasticity is solely dependent on agonist binding and occurs in the presence of co-agonist and ion-channel blockers.^10,24^ Recent studies from this laboratory, in which the functional and structural effects of brief exposure to NMDA were monitored simultaneously, confirmed that LTD and spine shrinkage mechanistically diverge.^24^ NMDAR-LTD was abolished in the presence of the ion-channel blocker MK-801 and by 7-CK, an antagonist at the co-agonist binding site, while spine shrinkage remained intact. Together, these findings indicate that NMDARs can signal in both an ionotropic and non-ionotropic mode to trigger LTD and spine shrinkage, respectively. Non-ionotropic NMDAR signaling was originally described as “metabotropic,”^14,25^ so we use the term “mNMDAR” to refer to this type of signaling.^24^

Further examination of mNMDAR signaling revealed that spine shrinkage was dependent on the activation of mammalian target of rapamycin complex 1 (mTORC1) and new protein synthesis.^24^ Dysregulated synaptic protein synthesis and altered spine structure are features of fragile X syndrome (FXS), an NDD characterized by ID and ASD and caused by loss of the fragile X messenger ribonucleoprotein (FMRP).^26–28^ This structural readout of mNMDAR function was investigated in the mouse model of FXS, whereby the magnitude of spine shrinkage in response to NMDA appeared intact. However, it proceeded in the presence of cycloheximide, consistent with the conclusion that the local protein synthesis that normally gates spine plasticity is dysregulated in this disorder.^24^

To further investigate mNMDAR signaling and its contribution to the pathophysiology in FXS, we have used spine shrinkage as an assay to dissect the GluN2 subtype and structural motifs that transduce agonist binding to the initiation of mNMDAR signaling. The data show that ligand binding to the GluN2B subunit is obligatory and that signaling is mediated via the GluN2B carboxy-terminal domain (CTD). Eliminating mNMDAR signaling by replacing the GluN2B CTD with the GluN2A CTD phenocopies several core aspects of FXS pathophysiology in the hippocampus, namely, increased density of dendritic spines, an elevated rate of bulk basal protein synthesis, exaggerated LTD mediated by G-protein-coupled metabotropic glutamate receptors (mGluR-LTD) in area CA1, and increased epileptiform activity in area CA3. Conversely, augmenting mNMDAR signaling pharmacologically—or by crossing *Fmr1* knockout (KO) mice with animals in which the GluN2A CTD is replaced with the GluN2B CTD—corrects protein synthesis, mGluR-LTD, and audiogenic seizure phenotypes. Together, the results suggest that GluN2B might be a novel therapeutic target in FXS.

## RESULTS

In hippocampal pyramidal neurons, NMDARs are tetramers consisting of two obligatory GluN1 and two GluN2 (A and B) subunits that co-assemble in to a di- or tri-heteromeric arrangement. Although GluN2B predominates at birth and is expressed throughout adulthood, GluN2A levels gradually increase during postnatal development and impose a distinct and dominant kinetic profile on the NMDAR assembly.^29,30^ Since GluN2A/2B subunits have varied distributions and physiological roles, an important objective is to determine the subtype specificity of mNMDAR signaling and understand how it elicits structural plasticity at CA1 synapses. Studying the respective contribution of the GluN2 subtype is complex because deletion of *Grin2B* is lethal from birth, and widespread loss leads to disruption in network activity.^31,32^ Therefore, in addition to utilizing a constitutive *Grin2A* KO mouse line, we employed genetically engineered mice carrying conditional KO alleles for GluN2A (*Grin2A^fl/fl^*) and GluN2B (*Grin2B^fl/fl^*) to determine the subtype specificity of mNMDAR signaling.

We simultaneously examined the contribution of the GluN2A subtype to NMDAR-dependent LTD and spine shrinkage in transverse hippocampal slices prepared from the progeny of *Grin2A* KO mice crossed with a Thy1-GFP line (postnatal days 25–35 [P25–35]). These mice express GFP in a random subset of neurons, and using two-photon time-lapse fluorescence imaging we were able to visualize dendritic spines along isolated apical dendrites of CA1 pyramidal neurons in stratum radiatum. In addition, we performed extracellular recordings by stimulating the Schaffer collateral pathway every 30 s and recorded the corresponding field excitatory postsynaptic potentials (fEPSPs) from the region surrounding the dendrite of interest. To induce NMDAR-dependent LTD and spine shrinkage, we briefly applied NMDA (20 μM, 3 min), to trigger a saturating and persistent reduction in the fEPSP slope and spine volume.^4,24^

### GluN2B triggers non-ionotropic NMDAR signaling and the retraction of dendritic spines

**Figure 1.**
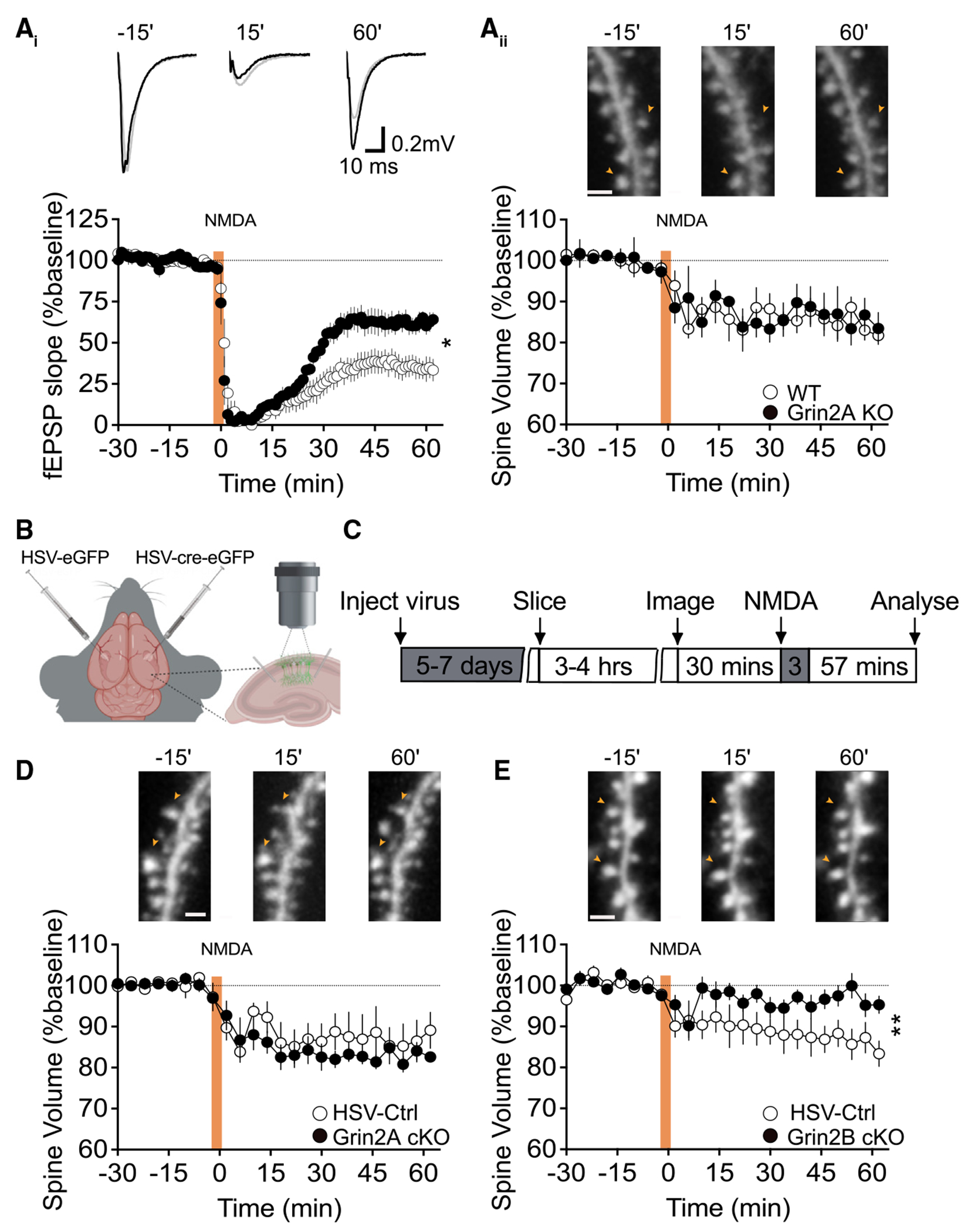
Spine structural plasticity triggered by non-ionotropic NMDAR signaling depends on the GluN2B subunit (A) Extracellular field recordings and time-lapse two-photon imaging were simultaneously performed in CA1 stratum radiatum of acute hippocampal slices prepared from *Grin2A* KO Thy1-GFP mice and WT littermates. NMDA infusion occurred during the time indicated by the orange shading. (A_i_) Brief bath application of NMDA (20 μM; 3 min) induces a persistent reduction in the slope of the fEPSP in WT Thy1-GFP slices that is significantly impaired in *Grin2A* KO Thy1-GFP slices (WT: 35% ± 6%, *n* = 8; *Grin2A* KO: 62% ± 5%, *n* = 5; *p = 0.01, unpaired t test). (A_ii_) In contrast, NMDA triggered a decrease in spine volume in WT Thy1-GFP slices that persisted in the absence of the *Grin2A* allele (WT: 84% ± 2%, *n* = 8; *Grin2A* KO: 85% ± 5%, *n* = 5; *p* = 0.87, unpaired t test), indicating that the GluN1/GluN2B di-heteromeric receptors are sufficient to support mNMDAR signaling. (B) Schematic of HSV injections into the dorsal hippocampus of floxed *Grin2A* and *Grin2B* mice. Created with bioRender.com. (C) Experimental timeline for the conditional knockout (cKO) of *Grin2A* and *Grin2B* in the dorsal hippocampus of floxed *Grin2A* and *Grin2B* mice through injections of either HSV-eGFP control or HSV-eGFP-Cre. (D) Conditional deletion of the *Grin2A* gene has no effect on structural plasticity in response to NMDAR activation (HSV control: 87% ± 5%, *n* = 4; HSV-Cre-*Grin2A* cKO: 83% ± 1%, *n* = 7; *p* = 0.36, unpaired t test). (E) Loss of *Grin2B* significantly impairs NMDA-induced spine shrinkage (HSV control: 86% ± 3%, *n* = 6; HSV-Cre-*Grin2B* cKO: 97% ± 2%, *n* = 10; *p = 0.0097, unpaired t test). Representative fEPSP traces (scale bar 10 ms/0.2 mV) and images (scale bar is 5 μm) in (A), (D), and (E) are shown 15 min before, 15 min after, and 60 min after LTD induction. Data presented as mean ± SEM.

We observed that the acute application of NMDA induced a persistent depression of fEPSP responses in wild-type (WT) Thy1-GFP slices that was significantly reduced, but not fully abolished, in *Grin2A* KO Thy1-GFP mice (Figure 1A_i_). In contrast, NMDA triggered a reduction in spine volume that was indistinguishable between *Grin2A* KO and WT Thy1-GFP slices (Figure 1A_ii_). These findings suggest that GluN2A-containing assemblies play a role in functional NMDAR-LTD but are not required for the induction of spine shrinkage. Therefore, the GluN2B di-heteromers may be sufficient to initiate the non-ionotropic signaling that underlies NMDA-induced structural plasticity at CA1 synapses.

To test this hypothesis, juvenile *Grin2A* and *Grin2B* floxed mice (P25) received a transcranial injection into the dorsal hippocampus of a herpes simplex virus (HSV) expressing cre-eGFP fusion protein or HSV-eGFP control (Figure 1B). After 5–7 days, hippocampal slices were prepared and imaged (Figure 1C). Consistent with previous reports, we observed sparse eGFP signal throughout CA1 indicating that the HSV-Cre-eGFP eliminated the floxed *Grin2A* or *Grin2B* allele in a small population of CA1 pyramidal neurons.^33^ In agreement with our findings from *Grin2A* KO Thy1-GFP mice, the acute application of NMDA led to a comparable reduction in spine volume in conditional *Grin2A^fl/fl^* and control mice (Figure 1D). However, conditional deletion of *Grin2B*, which spares only the GluN2A di-heteromeric receptors, abolished spine shrinkage at CA1 synapses (Figure 1E). Thus, the GluN2B-containing receptors are obligatory and sufficient to induce mNMDAR signaling. Together, these findings indicate that GluN2A- and GluN2B-containing di- and tri-heteromeric assemblies contribute differentially to NMDAR-dependent functional and structural plasticity at CA1 synapses.

### Pharmacology of NMDAR-mediated functional and structural plasticity indicates that agonist binding to GluN2B is critical for non-ionotropic signaling

**Figure 2.**
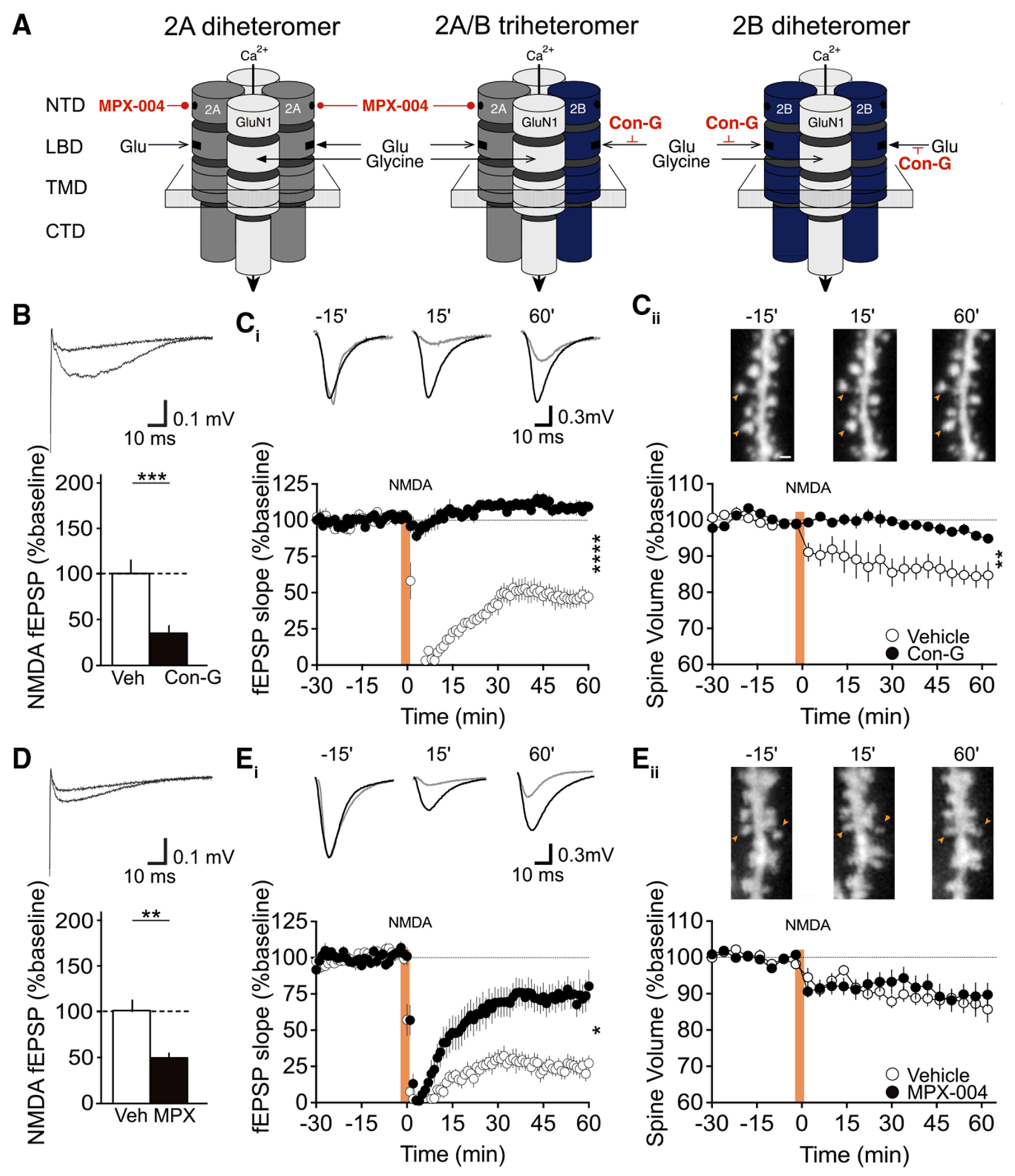
Pharmacology of NMDAR-mediated functional and structural plasticity indicates that agonist binding to GluN2B is critical (A) Schematic of the predominant NMDAR assemblies in hippocampal CA1 and binding sites for GluN2A and GluN2B subtype-selective compounds. The GluN2B competitive antagonist conantokin-G (Con-G) blocks the glutamate binding site on 2A/2B tri-heteromers and 2B di-heteromers. The negative allosteric modulator (NAM) of GluN2A, MPX-004, binds to the N-terminal domain (NTD) of 2A di-heteromers and 2A/2B triheteromers. (B and C) Con-G (2 μM) eliminated 66% of the isolated NMDAR-mediated fEPSP (B; WT vehicle: 100% ± 14%; WT Con-G: 34% ± 6%, *n* = 15; *p* = 0.00016, unpaired t test) and abolished both NMDA-induced LTD (C_i_; WT vehicle: 46% ± 6%, *n* = 8; WT Con-G: 108% ± 3%, *n* = 10; *p < 0.0001, unpaired t test) and spine shrinkage at CA1 synapses (C_ii_; WT vehicle: 85% ± 3%, *n* = 8; WT Con-G: 96% ± 1%, *n* = 9; *p = 0.002, unpaired t test). (D and E) MPX-004 (30 μM) caused a 47% block of the isolated NMDAR-fEPSP (D; WT vehicle: 100% ± 13%; WT MPX-004: 53% ± 4%, *n* = 14; *p = 0.0013, unpaired t test) and a comparable inhibition of LTD (E_i_; WT vehicle: 46% ± 7%, *n* = 11; WT MPX-004: 75% ± 11%, *n* = 13; *p = 0.03; unpaired t test), but had no effect on spine shrinkage (E_ii_; WT vehicle: 87% ± 3%, *n* = 11; WT MPX-004: 89% ± 3%, *n* = 13; *p* = 0.64, unpaired t test). Representative NMDAR-mediated fEPSP traces in (B) and (D) scale bar is 10 ms/0.1 mV. Representative fEPSP traces (scale bar 10 ms/0.3 mV) and images (scale bar is 2 μm) in (C) and (E) are shown 15 min before, 15 min after, and 60 min after LTD induction. Data presented as mean ± SEM.

NMDAR subunits have a similar topology consisting of three transmembrane domains (TMDs), one re-entrant loop that comprises the channel pore, an extracellular amino-terminal domain (NTD), a ligand-binding domain (LBD), and an intracellular CTD that couples the receptor to intracellular signaling messengers.^34^ The GluN2A and GluN2B subtypes show a high degree of similarity in their amino acid structures, particularly in their NTD and TMD, making it difficult to pharmacologically isolate these subunits.^35^ However, there are several compounds that show greater selectively toward GluN2A- or GluN2B-containing receptors (Figure 2A).

Here, we utilized conantokin-G, a competitive antagonist of the GluN2B LBD, and MPX-004, a negative allosteric modulator (NAM) of GluN2A containing NMDARs.^36,37^ We observed that blocking the GluN2B LBD with conantokin-G (2 μM), which leaves the LBD of GluN2A di-heteromers intact, eliminated 66% of the isolated NMDAR-mediated fEPSP (Figure 2B) and abolished both NMDA-induced LTD and spine shrinkage at CA1 synapses (Figures 2C_i_ and 2C_ii_). The LTD and spine findings are comparable to those using the non-selective, competitive antagonist D-APV.^24^ These data suggest that activation of GluN2A di-heteromers alone is not sufficient to drive either NMDA-dependent functional or structural plasticity at CA1 synapses and supports the conclusion from the conditional genetic studies that GluN2B is essential for the mNMDAR signaling that culminates in spine shrinkage.

Next, we pharmacologically targeted GluN2A using the NAM MPX-004 (30 μM), which strongly inhibits ion flux through the GluN2A di- and tri-heteromeric assemblies.^36,38^ We observed that MPX-004 caused a 47% block of the isolated NMDAR fEPSP (Figure 2D) and a comparable inhibition of LTD (Figure 2E_i_) but had no effect on spine shrinkage (Figure 2E_ii_). Together, the data suggest that GluN2A-containing receptors may substantially contribute to the induction of functional LTD, although spine shrinkage is solely dependent on agonist binding to the LBD of GluN2B-containing receptors.

Ro25-6981 binds to the NTD of GluN2B and limits ion flux through GluN2B di-heteromeric receptors, with minimal effect on GluN2A/B tri-heteromeric receptors.^39^ In our preparation, Ro25-6981 (30 μM) inhibited the NMDAR-mediated excitatory postsynaptic current (EPSC) by 51% but had no impact on the magnitude of either spine shrinkage or NMDA-induced LTD (Figure S1). These findings are consistent with the conclusion that NMDA-induced spine shrinkage is triggered by activation of the LBD of GluN2B di-heteromeric receptors. Only when this LBD is blocked using the GluN2B-selective competitive inhibitor conantokin-G do we see that spine shrinkage is fully abolished.

### NMDARs induce spine shrinkage through the GluN2B CTD

**Figure 3.**
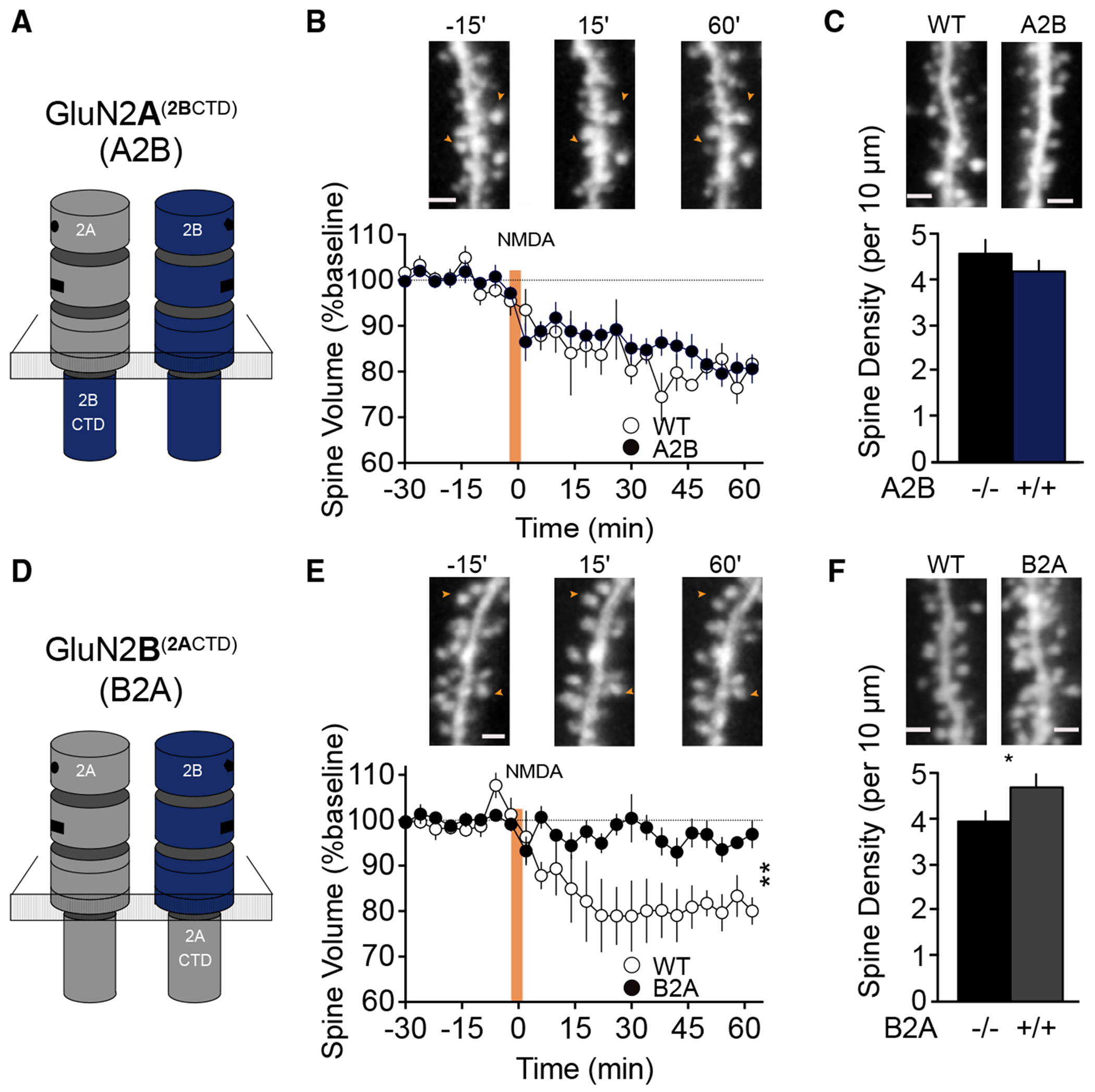
Non-ionotropic NMDARs induce spine shrinkage through the GluN2B carboxyl terminal domain (A and D) Schematics of genetically engineered GluN2A^2BCTD^ (termed A2B) and GluN2B^2ACTD^ (termed B2A) knockin mouse lines, where the exon encoding the CTD of GluN2A is deleted and replaced with GluN2B (A2B) and vice versa (B2A). (B and C) Introduction of the GluN2B CTD on GluN2A-containing receptors has no significant effect on NMDA-induced structural plasticity (B; WT: 80% ± 2%, *n* = 4; GluN2A^2BCTD^ HOM: 81% ± 3%, *n* = 6; *p* = 0.96, unpaired t test) or spine density along apical dendrites of CA1 pyramidal neurons (C; WT: 4.60 ± 0.64, *n* = 5; GluN2A^2BCTD^ HOM: 4.20 ± 0.21, *n* = 6; *p* = 0.25, unpaired t test). (E and F) Switching the GluN2B CTD with 2A leads to a significant impairment in NMDA-induced structural plasticity (E; WT: 81% ± 3%, *n* = 4; GluN2B^2ACTD^ HOM: 96% ± 2%, *n* = 6; *p = 0.005, unpaired t test) and a modest increase in spine density in B2A homozygous mice relative to WT littermates (F; WT: 3.96 ± 0.20, *n* = 5; GluN2B^2ACTD^ HOM: 4.72 ± 0.20, *n* = 7; *p = 0.029, unpaired t test). Representative images in (B) and (E) are from 15 min before, 15 min after, and 60 min after LTD induction. For (B), (C), (E), and (F) scale bar is 5 μm. Data presented as mean ± SEM.

As ligand binding alone can trigger mNMDAR signaling in the absence of ion flux, we hypothesized that it may be transduced through the CTD of GluN2B. While the NTDs and TMDs of GluN2A and GluN2B are highly conserved, their large intracellular CTDs exhibit considerable sequence heterogeneity.^35^ It has been previously reported that the GluN2B CTD is crucial for coupling the NMDAR assembly to a large complex of proteins and has distinct effects on synaptic plasticity and behavior relative to the GluN2A CTD.^40^ Thus, we speculated that the CTDs may also be the locus for the different roles played by GluN2B and GluN2A NMDAR assemblies in eliciting mNMDAR signaling and spine retraction. To test this idea we utilized a transgenic knockin line, where the exon encoding the CTD of GluN2A is deleted and replaced with the GluN2B CTD (GluN2A^2BCTD^, “A2B”; Figure 3A) and vice versa (GluN2B^2ACTD^, “B2A”; Figure 3D). Importantly, the NTD, LBD, and TMD are left intact in GluN2A^2BCTD^ and GluN2B^2ACTD^ mice, and switching the CTD has been reported to have no functional effect on NMDAR EPSCs or NMDA/α-amino-3-hydroxy-5-methyl-4-isoxazolepropionic acid receptor (AMPAR) ratios in the hippocampus.^40^ Crossing the GluN2A^2BCTD^ and GluN2B^2ACTD^ mice with the Thy1-GFP line enabled us to examine structural plasticity downstream of NMDAR activation. We observed that deleting the CTD of GluN2A and replacing it with GluN2B had no impact on spine shrinkage at CA1 synapses (Figure 3B). In contrast, eliminating the CTD of GluN2B and replacing it with GluN2A abolished spine shrinkage in GluN2B^2ACTD^ mice relative to WT littermates (Figure 3E). These findings suggest that NMDA triggers mNMDAR signaling through the GluN2B CTD of di-heteromeric assemblies.

During development, a failure to refine neural circuitry in an activity-dependent manner has been shown to lead to an overabundance of dendritic spines that yields excessive excitatory synapses.^41^ Therefore, we speculated whether there may be an abnormality in dendritic spine density in GluN2B^2ACTD^ mice due to the loss of NMDAR-induced spine shrinkage and elimination. To test this hypothesis, we quantified spine density along the apical dendrites of CA1 pyramidal neurons in GluN2A^2BCTD^ and GluN2B^2ACTD^ mice. We observed no significant difference in spine density between GluN2A^2BCTD^ and WT slices (Figure 3C), but in the absence of the 2B CTD there was a subtle, but significant, increase in spine density in GluN2B^2ACTD^ mice relative to WT controls (Figure 3F).

### GluN2B CTD bidirectionally regulates protein synthesis and mGluR-LTD

**Figure 4.**
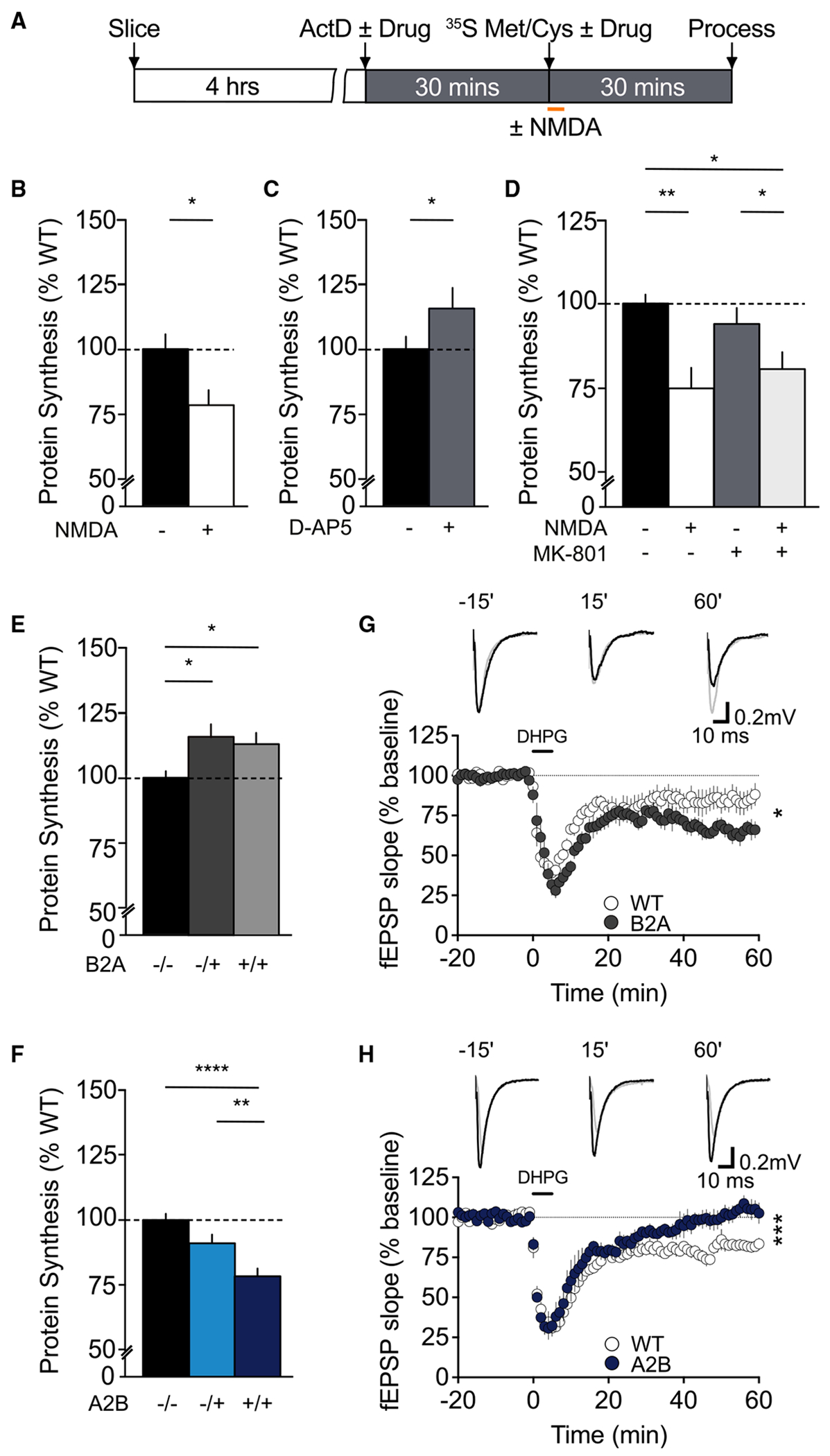
Non-ionotropic NMDAR signaling through the GluN2B CTD negatively regulates bulk protein synthesis and mGluR-LTD (A) Experimental timeline for measuring protein synthesis in acute hippocampal slices. Transverse hippocampal slices were recovered for 4 h in ACSF before transcription was blocked with actinomycin D (25 μM) ± drug, and newly synthesized proteins were radiolabeled with [^35^S]methionine/cysteine (10 μC_i_/mL) ± drug. (B) The acute application of NMDA (20 μM, 3 min) leads to a significant decrease in the ^35^S incorporation relative to vehicle-treated slices (vehicle: 100% ± 5%; NMDA: 79% ± 6%; *n* = 8; *p = 0.014, paired t test). (C) In contrast, the non-selective, competitive antagonist D-APV (50 μM) causes a significant increase in protein synthesis in hippocampal slices (vehicle: 100% ± 4%; D-APV: 117% ± 6%; *n* = 12; *p = 0.046, paired t test). (D) NMDA leads to a reduction in ^35^S incorporation relative to vehicle-treated slices, which persisted in the presence of the NMDAR ion-channel blocker MK-801 (vehicle: 100% ± 2%; NMDA: 75% ± 6%; MK-801: 97% ± 5%; NMDA/MK-801: 82% ± 5%; *n* = 7; *p = 0.0018, ANOVA). (E) Protein synthesis is elevated in GluN2B^2ACTD^ (B2A) mice compared to WT littermates (WT: 100% ± 2%, *n* = 13; B2A HET: 116% ± 5%, *n* = 7; B2A HOM: 113% ± 4%, *n* = 8; *p = 0.0051, ANOVA). (F) Genetic introduction of GluN2B CTD into the GluN2A locus (GluN2A^2BCTD^; A2B) reduces protein synthesis in a gene-dose-dependent manner (WT: 100% ± 2%, *n* = 13; A2B HET: 91% ± 3%, *n* = 15; A2B HOM: 79% ± 3%, *n* = 15; *p < 0.0001, ANOVA). (G) Acute application of the group 1 mGluR_1/5_ agonist DHPG (50 μM, 5 min) induces a long-lasting depression of fEPSPs in hippocampal CA1. Replacing the GluN2B CTD with GluN2A significantly enhanced mGluR-LTD in B2A mice relative to WT littermates (WT: 83% ± 5%, *n* = 7; B2A HOM: 65% ± 4%, *n* = 6; *p = 0.017, unpaired t test). (H) In contrast, mGluR-LTD is reduced in the hippocampus of A2B mice relative to WT littermates (WT: 80% ± 2%, *n* = 6; A2B HOM: 104% ± 4%, *n* = 5; *p = 0.0008, unpaired t test). Representative traces (scale bar 10 ms/0.2 mV) in (G) and (H) are shown at 15 min before, 15 min after, and 60 after LTD induction. Data presented as mean ± SEM.

Previously, we have shown that spine shrinkage is sensitive to the mRNA translational inhibitor cycloheximide and the mTORC1 inhibitor rapamycin. These findings suggested that mNMDAR signaling may trigger *de novo* protein synthesis via the mTORC1 pathway to support the persistent reduction in spine volume.^24^ To directly measure mRNA translation, we performed a metabolic labeling assay in acute hippocampal slices that mimics conditions used to record long-term changes in functional and structural plasticity. Hippocampal slices were recovered in oxygenated artificial cerebrospinal fluid (ACSF) for 4 h to allow metabolic processes to recover. Transcription was then blocked with actinomycin D (25 μM) for 30 min to isolate mRNA translation before newly synthesized proteins were labeled with [^35^S] methionine/cysteine protein labeling mix (Figure 4A). To determine whether the activation of NMDARs can drive changes in bulk protein synthesis, we stimulated slices with NMDA (20 μM, 3 min) or vehicle during protein labeling before transferring slices to ACSF containing [^35^S]methionine/cysteine for a further 27 min. We observed that NMDA caused a significant reduction in ^35^S incorporation (Figure 4B), whereas incubating hippocampal slices with the competitive antagonist D-APV (50 μM), 30 min prior and throughout protein labeling, led to a significant increase in mRNA translation (Figure 4C). These data demonstrate that targeting the LBD of NMDARs can bidirectionally regulate global protein synthesis rates in the hippocampus.

Based on these findings, we wanted to determine whether NMDARs can alter mRNA translation in the absence of ion flux. Hippocampal slices were incubated in ACSF containing actinomycin D ± MK-801 and labeled with [^35^S]methionine/cysteine ± MK-801. During metabolic labeling, slices were briefly stimulated with NMDA or vehicle ± MK-801. We observed that NMDA causes a significant reduction in ^35^S incorporation in the presence of MK-801 (Figure 4D). These findings suggest that NMDARs can alter translational rates in the absence of ion permeation to support spine shrinkage at CA1 synapses. Although it may seem paradoxical that bulk protein synthesis is decreased by stimulating the mTORC1 pathway through GluN2B, these findings are consistent with previous studies in the *Tsc2* heterozygous mouse^42^ and reflect a change in the balance between pools of mRNA competing for access to ribosomes.^43^

Encouraged by these findings, we next wanted to examine whether switching the GluN2A and GluN2B CTD could influence protein synthesis rates under basal conditions in the absence of exogenous stimulation. We observed that deleting the GluN2B CTD and replacing it with GluN2A (GluN2B^2ACTD^/B2A mice) led to a significant increase in protein synthesis relative to WT slices (Figure 4E). Conversely, eliminating the GluN2A CTD and replacing it with 2B (GluN2A^2BCTD^/A2B mice) significantly reduced protein synthesis in GluN2A^2BCTD^ heterozygous and homozygous mice in a gene-dose-dependent manner (Figure 4F). Together, these findings suggest that the GluN2 CTD regulates basal protein synthesis levels in the hippocampus.

One form of synaptic plasticity that is sensitive to alterations in protein synthesis is mGluR-LTD.^44^ Increases in bulk protein synthesis, observed in mouse models of FXS and *Syngap1* haploinsufficiency, coincide with an elevation in mGluR-LTD that is no longer gated by new protein synthesis.^27,45,46^ In contrast, decreases in bulk protein synthesis, as observed in *Tsc2* heterozygous mice, lead to an impairment in mGluR-LTD.^42^ Together they define an axis of synaptic pathophysiology, where deviations in protein synthesis can be used to predict disease related cellular abnormalities. Therefore, we hypothesized that protein-synthesis-dependent phenotypes may be disrupted in GluN2B^2ACTD^ and GluN2A^2BCTD^ mice, mimicking *Fmr1* KO and *Tsc*2 heterozygous mice, respectively.

To test this hypothesis, we examined mGluR-LTD in GluN2B^2ACTD^ and GluN2A^2BCTD^ mice. Acute hippocampal slices were prepared and recovered for 3–4 h before extracellular recordings were performed in CA1. mGluR-LTD was induced by acute application of the mGluR_1/5_ agonist dihydroxyphenylglycine (DHPG; 50 μM, 5 min^47^), and the slopes of fEPSPs were monitored for a further 55 min. DHPG caused a significant reduction of fEPSP responses in WT slices that was exaggerated in GluN2B^2ACTD^ mice (Figure 4G). In contrast, the magnitude of mGluR-LTD was significantly reduced in GluN2A^2BCTD^ mice (Figure 4H). Thus, GluN2B^2ACTD^ and GluN2A^2BCTD^ show mirror-opposite alterations in mGluR-LTD relative to WT littermates, reminiscent of findings observed in mouse models of FXS and tuberous sclerosis complex (TSC), respectively. The finding that the maximal transient depression immediately following application of DHPG did not differ between GluN2 CTD genotypes suggests that these findings are unlikely to be accounted for by differences in mGluR_5_ expression.^48^

### Loss of mNMDAR signaling promotes epileptogenesis in the hippocampus

**Figure 5.**
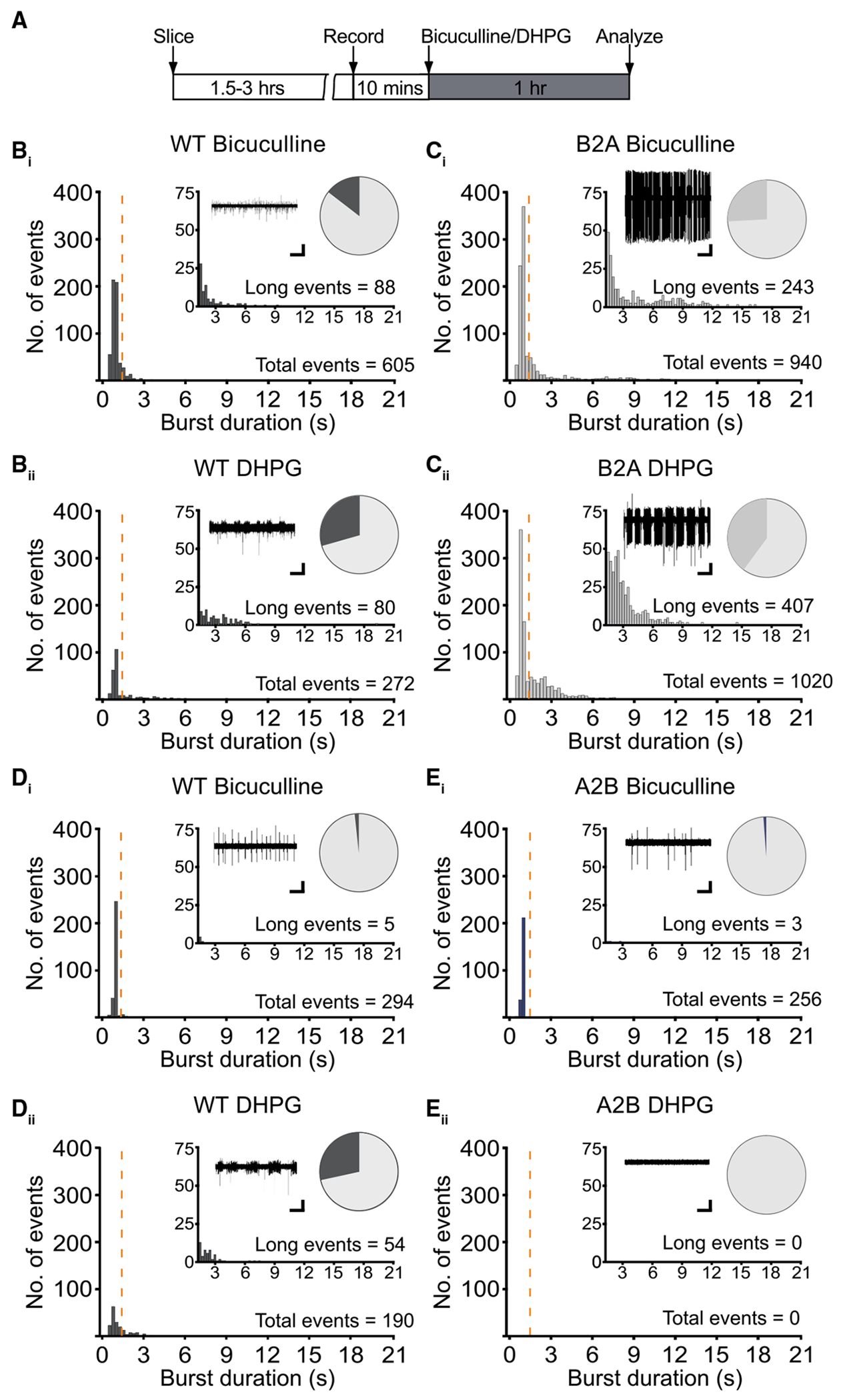
Enhancing or eliminating mNMDAR signaling by switching the GluN2 CTD bidirectionally regulates CA3 epileptogenesis (A) Experimental timeline for extracellular recordings in hippocampal CA3. Transverse hippocampal slices were recovered before a recording electrode was placed in the pyramidal layer of CA3. Slices were perfused with ACSF for 10 min before being treated with either the GABA_A_ receptor antagonist bicuculline (Bic, 50 μM) or the group 1 mGluR agonist DHPG (50 μM) for 1 h. (B) (B_i_) Extracellular recordings in CA3 pyramidal cell layer of WT hippocampal slices perfused with ACSF containing bicuculline primarily generates short rhythmic events (short events <1.5 s = 517, long events ≥1.5 s = 88, average burst duration of 605 events = 1.23 ± 0.04 s, *n* = 8). (B_ii_) Bath application of DHPG generates significantly longer burst events (short events <1.5 s = 192, long events ≥1.5 s = 80, average burst duration of 272 events = 1.76 ± 0.10 s, *n* = 11; *p* < 0.0001, ANOVA). (C) (C_i_) GABA_A_ blockade with bicuculline triggers a greater number of epileptiform discharges that are longer in burst duration in CA3 region of hippocampal slices from GluN2B^2ACTD^ (B2A) mice compared to WT mice (B2A: short events <1.5 s = 697, long events ≥1.5 s = 243, average burst duration of 940 events = 1.88 ± 0.07 s, *n* = 7; *p < 0.0001, ANOVA). (C_ii_) Similarly, burst frequency and duration are increased in B2A slices perfused with DHPG compared to WT mice (B2A: short events <1.5 s = 613, long events ≥1.5 s = 407, average burst duration of 1,020 events = 1.83 ± 0.05 s, *n* = 7; *p < 0.0001, ANOVA). (D) (D_i_) WT hippocampal slices perfused with ACSF containing bicuculline primarily generates short rhythmic events (bicuculline: short events <1.5 s = 289, long events ≥1.5 s = 5, average burst duration of 294 events = 1.04 ± 0.01 s, *n* = 9). (D_ii_) Bath application of DHPG generates short rhythmic events that lead to an increase in prolonged discharges (DHPG: short events <1.5 s = 136, long events ≥1.5 s = 54, average burst duration of 190 events = 1.42 ± 0.07 s, *n* = 10). (E) (E_i_) Bicuculline induces comparable short and long events in GluN2A^2BCTD^ (A2B) mice compared to WT littermates (A2B: short events <1.5 s = 253, long events ≥1.5 s = 3, average burst duration of 256 events = 1.03 ± 0.01 s, *n* = 8). (E_ii_) DHPG can no longer induce short or prolonged discharges in A2B hippocampal slices (A2B: short events <1.5 s = 0, long events ≥1.5 s = 0, *n* = 7). Data presented as mean ± SEM. All scale bars, 30 s and 50 μV.

Previous studies have shown that elevation of basal protein synthesis can lead to epileptogenesis within area CA3 of the hippocampus,^49^ which can be mimicked by stimulating mGluR_1/5_ with DHPG. To examine whether the GluN2 CTD modulates excitability, we placed a recording electrode in the pyramidal layer of CA3 in transverse hippocampal slices from GluN2B^2ACTD^, GluN2A^2BCTD^, and WT littermates. Slices were perfused with ACSF for 10 min to obtain a baseline before we monitored spontaneous and epileptiform activity in response to the GABA_A_ antagonist bicuculline (50 μM) or DHPG (50 μM), respectively (Figure 5A). In control slices, addition of bicuculline led to short, rhythmic, ictal-like events of <1.5 s (Figure 5B_i_). However, in the presence of DHPG, ictal-like events progressed to prolonged synchronized discharges (Figure 5B_ii_). Intriguingly, in slices from GluN2B^2ACTD^ mice, GABA_A_ blockade alone led to a dramatic increase in burst duration and frequency (Figure 5C_i_), similar to the effect of DHPG (Figure 5C_ii_). In GluN2A^2BCTD^ slices, however, bicuculline had an effect similar to that seen in WT slices (Figures 5D and 5E_i_), and DHPG failed to induce short- or long-duration events (Figures 5D and 5E_ii_). Therefore, replacing the GluN2A CTD with the GluN2B CTD blocks mGluR_1/5_-induced epileptogenesis. Together, these data suggest that switching the GluN2 CTD leads to a hypersensitive and hyposensitive response to mGluR_1/5_ activation in GluN2B^2ACTD^ and GluN2A^2BCTD^ slices, respectively.

### Switching the GluN2A CTD with GluN2B normalizes protein synthesis and mGluR-LTD in the *Fmr1* KO mouse

**Figure 6.**
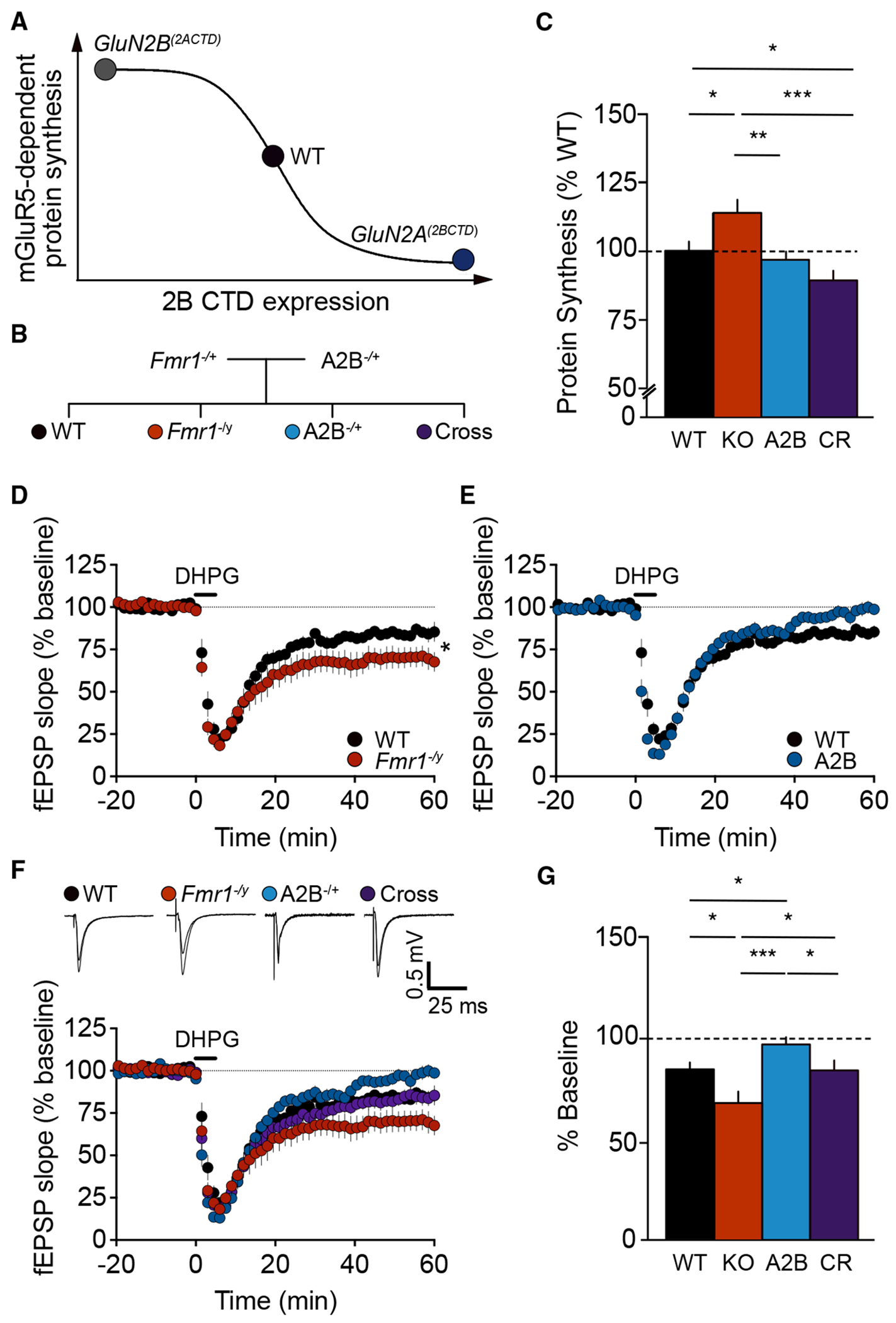
Increasing the expression of GluN2B C-terminal domain normalizes protein synthesis and mGluR-LTD in the *Fmr1* KO mouse (A) Schematic depicting the inverse relationship between GluN2B CTD expression and mGluR_5_ function. (B) Genetic rescue strategy in which *Fmr1* heterozygous females are crossed with male GluN2A^2BCTD^ (A2B) heterozygous mice to produce male offspring of four genotypes: wild type (WT), *Fmr1* KO (KO), A2B het (A2B), and *Fmr1* KO/A2B het cross (CR). (C) Replacing the C-terminal domain of GluN2A with GluN2B in *Fmr1* KO mice reduces elevated protein synthesis rates in the hippocampus (WT: 100% ± 3%, *n* = 21; *Fmr1* KO: 114% ± 5%, *n* = 11; A2B HET: 97% ± 3%, *n* = 13; cross: 89% ±3%, *n* = 14; A2B genotype *p = 0.0002, ANOVA). (D) The magnitude of DHPG-induced mGluR-LTD (50 μM, 5 min) in hippocampal CA1 is exaggerated in *Fmr1* KO mice relative to WT littermates (WT: 85% ± 3%, *n* = 10; *Fmr1* KO: 70% ± 6%, *n* = 10; *p = 0.029, unpaired t test). (E) In contrast, mGluR-LTD is significantly impaired in GluN2A^(2BCTD)^ heterozygote mice (WT: 85% ± 3%, *n* = 10; A2B: 98% ± 3%, *n* = 8; *p = 0.013, unpaired t test). (F) Introduction of the GluN2A^(2BCTD)^ mutation into *Fmr1* KO mice restores elevated mGluR-LTD to WT levels (WT: 85% ± 3%, *n* = 10; cross: 85% ± 4%, *n* = 9; *p* = 0.99, unpaired t test). Representative fEPSP traces (scale bar 25 ms/0.5 mV) are shown 15 min before and 60 min after LTD induction. (G) Comparison of all four genotypes reveals a suppressive effect of the GluN2A^(2BCTD)^ heterozygous mutation, lowering mGluR-LTD in the *Fmr1* KO hippocampus to WT levels (A2B/*Fmr1* genotype *p = 0.003, ANOVA). Data presented as mean ± SEM.

Based on these findings, it appears that GluN2B CTD expression strongly modulates functions related to mGluR_5_-dependent protein synthesis (Figure 6A). Deleting the GluN2B CTD in GluN2B^2ACTD^ mice exacerbates phenotypes downstream of mGluR_5_ activation, while genetically enhancing the expression of GluN2B CTD suppresses mGluR_5_-dependent phenotypes in the hippocampus. As there are numerous reports that mGluR_5_ contributes to disease phenotypes in mouse models of ID,^27,28,50^ we wanted to explore whether manipulating mNMDAR signaling in the *Fmr1* KO mouse could correct some of the core phenotypes in FXS. We introduced the GluN2A^2BCTD^ mutation into *Fmr1* KO mice by crossing *Fmr1* heterozygous females with GluN2A^2BCTD^ heterozygous males to enhance mNMDAR signaling (Figure 6B) and measured protein synthesis in the progeny. The results reveal that elevated protein synthesis in *Fmr1* KO mice is significantly reduced to WT levels by switching the CTD of GluN2A with GluN2B (Figure 6C).

The correction of protein synthesis with the introduction of the GluN2A^2BCTD^ knockin mutation led us to speculate that other pathological phenotypes could be reversed by augmenting GluN2B signaling in *Fmr1* KO mice. To examine this, we measured mGluR-LTD in the *Fmr1* KO × GluN2A^(2BCTD)^ cross. Consistent with previous studies, the acute application of DHPG (50 μM, 5 min) revealed that mGluR-LTD is exaggerated in *Fmr1* KO mice relative to WT littermates (Figure 6D). The GluN2A^(2BCTD)^ heterozygous mutation led to a small but significant suppression of mGluR-LTD when compared to WT mice (Figure 6E). Importantly, the *Fmr1* KO × GluN2A^2BCTD^ cross exhibits a magnitude of LTD indistinguishable from that in WT mice (Figures 6F and 6G). Together, our results show that the enhancement of GluN2B CTD expression through the introduction of the GluN2A^2BCTD^ mutation is sufficient to correct excessive protein synthesis and exaggerated mGluR-LTD in the *Fmr1* KO hippocampus.

### Dysregulated protein synthesis and audiogenic seizures in *Fmr1* KO mice are corrected by modulating GluN2B

**Figure 7.**
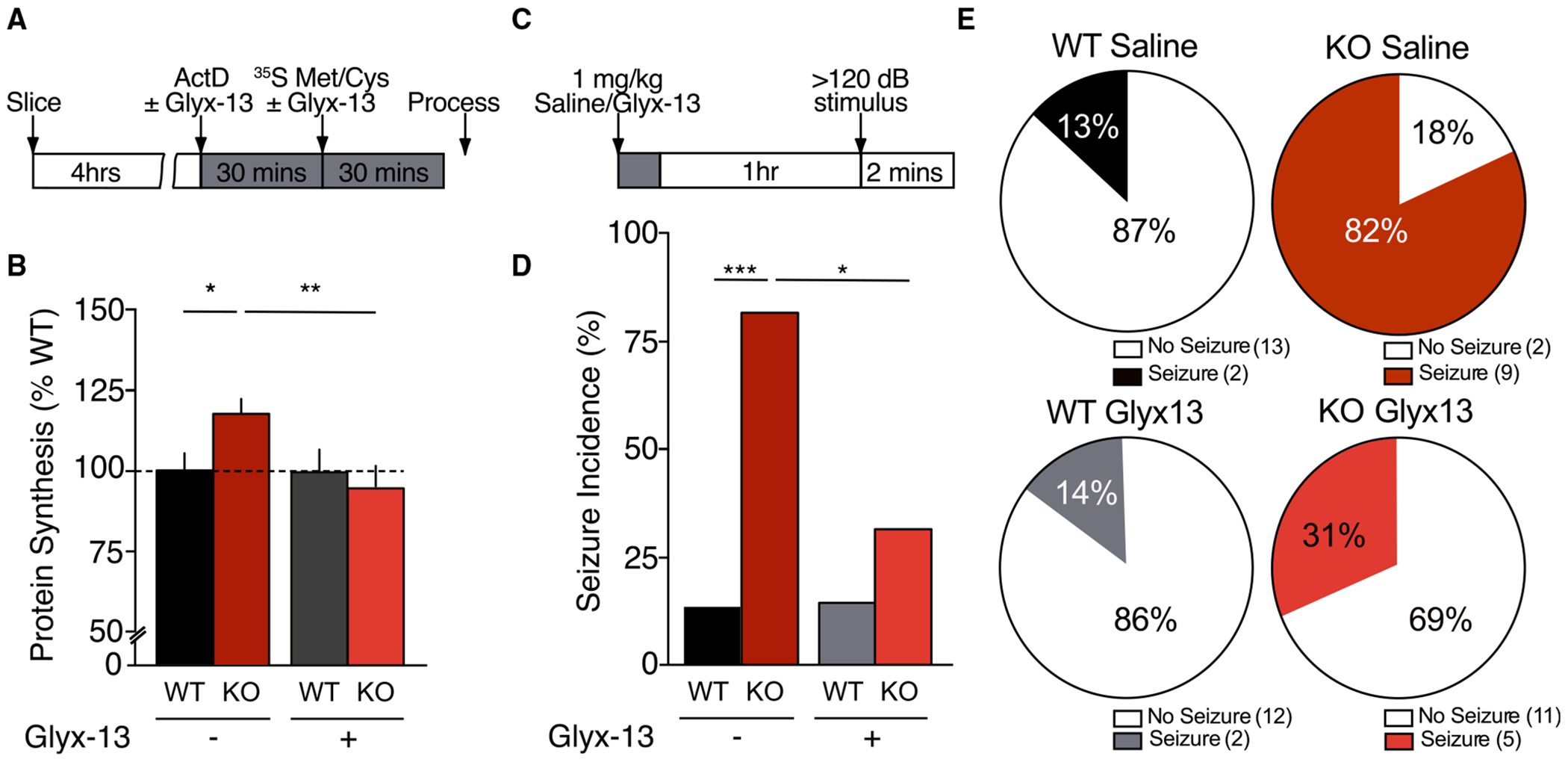
Dysregulated protein synthesis and audiogenic seizures in *Fmr1* KO mice are corrected by modulation of mNMDAR signaling (A) Experimental timeline for metabolic labeling in the presence of the NMDAR partial agonist Glyx-13. (B) Glyx-13 (0.1 μM) restored elevated protein synthesis in *Fmr1* KO mice to WT levels (WT vehicle: 100% ± 6%; KO vehicle: 118% ± 5%; WT Glyx-13: 99% ± 7%; KO Glyx-13: 94% ± 5%; *n* = 8; *p = 0.034, ANOVA). Data presented as mean ± SEM. (C) Experimental timeline for the *in vivo* treatment of WT and *Fmr1* KO mice with Glyx-13 (1 mg/kg) prior to audiogenic seizure (AGS) testing. Mice were injected with either saline or Glyx-13 (1 mg/kg) 1 h prior to AGS testing. Seizures were scored for wild running, clonic seizure, tonic seizure, and death. (D and E) AGS incidence is significantly increased in saline-treated *Fmr1* KO mice relative to WT littermates (WT saline: 13%, *n* = 15; KO saline: 82%, *n* = 11; *p = 0.0009, Fisher’s exact test). Treatment with Glyx-13 significantly reduced AGS incidence in *Fmr1* KO mice (KO saline: 82%, *n* = 11; KO Glyx-13: 31%, *n* = 16; *p = 0.018, Fisher’s exact test).

Previous studies in *Fmr1* KO mice have shown that correction of altered protein synthesis is highly predictive of improvements in structural, circuit, and behavioral phenotypes.^28,43,50,51^ According to our model, augmenting GluN2B signaling should ameliorate FXS phenotypes. Glyx-13 has recently been shown to preferentially target GluN2B-containing receptors at an allosteric site distinct from the glutamate- and glycine-binding sites.^52,53^ Furthermore, it is known to be a cognitive enhancer in several learning and memory paradigms.^54–56^ To test the potential utility of augmenting GluN2B function in FXS, we treated *Fmr1* KO and WT hippocampal slices with Glyx-13 (0.1 μM) and measured protein synthesis levels (Figure 7A). Once again, we observed a significant increase in ^35^S incorporation in *Fmr1* KO slices relative to WT littermates under basal conditions. Importantly, pretreatment with Glyx-13 normalized excessive protein synthesis in *Fmr1* KO mice to WT levels under these conditions (Figure 7B). This result supports the hypothesis that positively modulating GluN2B under tonic conditions corrects one of the core deficits in *Fmr1* KO mice.

In addition to an increase in spine density and hyperexcitability within the hippocampal circuit, another consequence of altered protein synthesis in *Fmr1* KO mice is enhanced susceptibility to audiogenic seizures (AGSs).^28,50,51^ Encouraged by our initial findings, we next wanted to determine whether Glyx-13 could reduce the incidence of AGS in *Fmr1* KO mice *in vivo*. *Fmr1* KO and WT littermates (P19–25) mice were injected with either saline or Glyx-13 (1 mg/kg). After 1 h, mice were transferred to a chamber and habituated for 1 min before they were exposed to a 120-dB stimulus for 2 min (Figure 7C). As expected, saline-treated *Fmr1* KO mice exhibited increased susceptibility to seizures relative to WT littermates (Figures 7D and 7E). However, a single dose of Glyx-13 was able to significantly reduce seizure susceptibility in *Fmr1* KO mice without impacting the incidence of seizures in WT mice. These findings further support the hypothesis that positively modulating GluN2B signaling can rescue both *in vitro* and *in vivo* phenotypes in *Fmr1* KO mice.

## DISCUSSION

The current study reveals that GluN2B is necessary and sufficient for mNMDAR signaling in the absence of ion flux and that this signaling can mitigate core FXS phenotypes linked to altered protein synthesis regulation. In addition, the data clarify the roles of GluN2A and GluN2B subunits in initiating NMDAR-dependent LTD and structural plasticity in CA1. A model accounting for the key findings is provided in Figure S2.

### NMDAR molecular motifs involved in non-ionotropic structural and ionotropic functional plasticity in CA1

We have previously shown that changes in dendritic spine volume in response to NMDA proceed independently of ion flux and are dependent on mTORC1 and *de novo* protein synthesis.^24^ Conversely, NMDAR-LTD occurs in the absence of mTORC1 activity or new protein synthesis but requires ion flux through the receptor channel. In the current study, we find that structural and functional plasticity in CA1 can be further dissociated by its dependence on GluN2 subunits. LTD, as measured by a change in synaptic transmission evoked by stimulation of the Schaffer collateral pathway, requires ligand binding to GluN2B but is also sensitive to genetic and pharmacological inhibition of GluN2A. Conversely, non-ionotropic signaling as reported by spine shrinkage is solely dependent on receptors containing GluN2B. We note that these conclusions explicitly apply to ages and experimental conditions when LTD depends on ion flux through the NMDARs and may differ during early development.^21^

We found that pharmacologically limiting ion flux through GluN2A-containing assemblies, or deleting the *Grin2A* gene, reduces the magnitude of NMDAR-LTD. However, LTD was fully abolished only when the LBD of GluN2B was blocked. Combined, these data suggest that both the GluN2B/GluN1 di-heteromeric and GluN2A/GluN2B/GluN1 tri-heteromeric assemblies contribute to LTD at CA1 synapses, whereas GluN2A/GluN1 di-heteromeric receptors do not. The GluN2B subunit is also crucial for non-ionotropic signaling reported as spine shrinkage. Structural plasticity was eliminated by antagonizing the LBD of GluN2B, genetically deleting this receptor subunit, or swapping out its CTD. Indeed, because deletion of GluN2A has no quantitative effect on spine shrinkage in response to NMDA, the data suggest that the GluN2B di-heteromeric receptors can account for the full effect of mNMDAR signaling.

Previous work has implicated CAMKII, p38 MAPK, and neuronal nitric oxide synthase signaling in driving actin depolymerization and the retraction of dendritic spines^10,12^ as well as gating spine growth associated with LTP.^13^ Our results indicate that GluN2B di-heteromeric assemblies are sufficient to support spine shrinkage caused by mNMDAR activation and that the GluN2B LBD and CTD are critical structural motifs. Considered with the previous study by Thomazeau et al.,^24^ who also used NMDA to trigger spine shrinkage, the data indicate that GluN2B signals through mTORC1 to modulate protein synthesis that gates structural plasticity.

How might the GluN2B CTD mediate its non-ionotropic functions and couple to downstream pathways controlling spine morphology and translation? Biochemical and genetic studies have demonstrated that the GluN2B CTD plays an essential role in the supramolecular assembly of NMDARs with scaffold proteins and signaling enzymes, which are key regulators of structural and functional plasticity.^57^ GluN2B di- and tri-heteromers assemble into 1.5-MDa supercomplexes, whereas GluN2A di-heteromers can only assemble into 0.8-MDa complexes lacking membrane-associated guanylate kinases (MAGUKs) and signaling molecules. Thus, the physiological functions observed for the GluN2B CTD could arise from its role in positioning of the receptor with its effector proteins and/or direct regulation of the signaling complex.

Although GluN2A signaling appears unnecessary for triggering spine shrinkage, there is evidence for non-ionotropic signaling by GluN2A/GluN1 di-heteromeric NMDARs leading to tyrosine dephosphorylation of the GluN2A subunit.^58^ Similarly, in HEK293 cells expressing GluN2A/GluN1 and in cultured cortical neurons, it has been reported that glycine triggers non-ionotropic signaling that confers neuroprotection.^59^ These and related studies indicate that non-ionotropic NMDAR signaling is multifaceted and not restricted to the regulation of spine structure by GluN2B in the hippocampus.^18,60^

### Regulation of protein synthesis by mNMDAR signaling

Further examination of mNMDAR signaling reveals that the GluN2B subtype can bidirectionally regulate protein synthesis. Brief NMDA application reduces bulk protein synthesis, and antagonizing the NMDAR LBD under basal conditions causes a significant elevation. Importantly, modulation of translational rates occurs in the presence of the channel blocker MK-801, suggesting that non-ionotropic mNMDAR signaling represents an important pathway by which synaptic activity regulates protein synthesis.

Consistent with this conclusion, we find that augmenting mNMDAR signaling by replacing the GluN2A CTD with the GluN2B CTD mimics the effect of agonist application on protein synthesis in WT hippocampus. Conversely, elimination of mNMDAR signaling by swapping out the GluN2B CTD mimics the effect of D-APV on WT protein synthesis. The functional significance of protein synthesis modulation by mNMDAR signaling through GluN2B is suggested by the impact on LTD induced by activating mGluR_1/5_ with DHPG.^47^ In the age range examined in this study, DHPG stimulates new protein synthesis that is required for induction of mGluR-LTD in WT mice.^28,44,47,61^ DHPG-induced mGluR-LTD is augmented in slices from GluN2B^(2ACTD)^ mice with increased protein synthesis rates, and it is reduced in slices from GluN2A^(2BCTD)^ mice with reduced basal protein synthesis. Going forward, it will be of great interest to investigate how the well-known effects of age and sensory experience on NMDAR subunit composition impact protein synthesis regulation and its functional consequences during development.^62^

Our findings of reduced global protein synthesis in response to mNMDAR activation are consistent with earlier reports in cortical cultures, which demonstrated that the GluN2B subunit suppresses protein synthesis.^63,64^ Interestingly, global protein synthesis is also reduced in a mouse model of TSC in which mTORC1 signaling is hyperactive.^42^ In this case, suppression of overall protein synthesis reflects a decrease in translation of terminal oligopyrimidine (TOP) containing mRNA transcripts and an increase in FMRP-binding targets that typically encode large synaptic proteins.^65^ When considered with the finding that mNMDAR-induced structural plasticity is blocked by the mRNA translation inhibitor cycloheximide, these results suggest that GluN2B triggers a shift in the translation pool rather than stalling mRNA translation per se. Based on these findings, we propose that agonist binding to the GluN2B LBD triggers a conformational change in the CTD of GluN2B di-heteromers to activate mTORC1 signaling and initiate mRNA translation and the synthesis of proteins that support the depolymerization of the actin cytoskeleton and the retraction of dendritic spines. It will be of great interest to identify actively translating mRNAs downstream of non-ionotropic mNMDAR signaling and understand how they contribute to structural plasticity at CA1 synapses.

### Mimicry and correction of core cellular phenotypes in *Fmr1* KO mice

Recently, it has been reported that elevated protein synthesis in *Fmr1* KO mice reflects a length-dependent shift in the translating pool of mRNAs.^66^ There is an overtranslation of short mRNA transcripts encoding ribosomal proteins at the expense of longer mRNAs encoding synaptic proteins and FMRP targets. Consequently, synaptic and circuit plasticity that is normally dependent on *de novo* protein synthesis are disrupted in the *Fmr1* KO and persist in the presence of protein synthesis inhibitors.^24,45,67^ We previously found that this also applies to NMDA-induced spine shrinkage in *Fmr1* KO mice, which, unlike WT, proceeds in the presence of cycloheximide.^24^

Here, we show that replacing the GluN2B CTD with GluN2A, thus eliminating mNMDAR signaling, leads to increased protein synthesis and spine density, exaggerated mGluR-LTD in CA1, and epileptiform activity in CA3. These differences in hippocampal structure and function phenocopy observations in the *Fmr1* KO mouse.^27,51,68^ An exception is spine shrinkage in response to NMDA, which is absent in B2A mice but present in *Fmr1* KO mice.^24^

On the flip side, overexpression of the GluN2B CTD reduces both LTD and the increase in neuronal excitability triggered by mGluR_1/5_ activation. Collectively, these data demonstrate that non-ionotropic mNMDAR signaling strongly modulates the intracellular consequences of activating mGluR_1/5_, which have been linked to pathophysiology in several NDDs that, in addition to fragile X, include TSC,^42,69^ *SYNGAP1* haploinsufficiency,^46^ Rett syndrome,^70^ chromosome 16p11.2 microdeletion syndrome,^71^ and autism.^72^

Numerous prior studies have shown that selectively inhibiting mGluR_5_ or its downstream signaling normalizes protein synthesis and rescues a multitude of synaptic and behavioral phenotypes in *Fmr1* KO.^73^ Similarly, enhancing mTORC1 signaling through the introduction of the *Tsc2* mutation corrects synaptic pathophysiology in FXS.^42^ This motivated us to try to reverse the core pathophysiology associated with *Fmr1* KO mice by increasing mNMDAR signaling through the introduction of GluN2A^2BCTD^ mutation. In the absence of FMRP, increasing the expression of the GluN2B CTD reduced exaggerated protein synthesis, mGluR-LTD, and epileptiform activity in the hippocampus of *Fmr1* KO mice.

Allosteric augmentation of mNMDARs, in principle, could improve synaptic regulation of mTORC1 and by doing so restore the normal balance of high- and low-efficiency mRNA translation in FXS.^74,75^ In a preliminary test of this hypothesis, we observed that Glyx-13, a positive modulator of NMDARs,^55,76^ corrected the protein synthesis phenotype in *Fmr1* KO hippocampus. Encouraged by these findings, we also investigated the consequence of modulating NMDARs on AGS, an *in vivo* phenotype in *Fmr1* KO mice that is associated with altered protein synthesis regulation.^77^ A single dose of Glyx-13^78^ was sufficient to significantly reduce the incidence of seizures. Glyx-13 acts on NMDARs at an allosteric site distinct from the glutamate- and glycine-binding sites.^52^ It remains to be determined whether the effect of this compound on AGS is independent of effects on NMDAR ion flux.

Mutations in GluN2B have been linked to numerous types of epilepsy including epileptic encephalopathies, focal epilepsy, partial seizures, and infantile spasms.^79–81^ In addition, deletion of GluN2B leads to an increase in the number of excitatory inputs onto pyramidal neurons in the prefrontal cortex.^64^ In the current study, we also detected an increase in CA1 spine density in the absence of the GluN2B CTD and, in CA3, we found prolongation of epileptiform bursts in response to mGluR_1/5_ activation. Together, these findings demonstrate that the GluN2B subunit makes a critical contribution to circuit refinement, protein synthesis regulation, and circuit excitability in the juvenile hippocampus. Identifying compounds that can selectively modulate this unique mode of signaling may prove to be a highly valuable target in treating NDDs.

### Limitations of the study

Most of our experiments were performed in P25–35 mice, consistent with previous work.^24^ We also examined NMDA-induced spine shrinkage, in the presence of MK-801, in a small cohort of mice ≥P60 and observed that NMDA induces a reduction in spine volume (83% ± 1.7%, *n* = 3), similar in magnitude to that observed in juvenile mice (P25–35). This is not surprising given that GluN2B remains the dominant subtype even as GluN2A expression levels increase.^40,82^ Nevertheless, we cannot rule out the possibility that mNMDAR signaling may be different in fully mature hippocampal synapses. This will require further study.

Numerous studies have shown that correction of aberrant protein synthesis in *Fmr1* KO (e.g., mGluR_5_ NAMs, lovastatin, metformin, GSK3 inhibitors) rescues an array of electrophysiological and behavioral phenotypes that include inhibitory avoidance deficits and audiogenic seizures.^73^ Although behavioral phenotypes were not studied in *Fmr1* KO × GluN2A^2BCTD^ double mutants, we did show that an NMDAR PAM, Glyx-13, rescues the seizure phenotype in *Fmr1* KOs. It will be of great interest to examine the effects of modulating mNMDAR signaling on additional behavioral phenotypes in *Fmr1* KO in future studies.

Bulk protein synthesis is reduced by mNMDAR activation in WT mice (Figure 4D). The findings that protein synthesis is elevated by the competitive LBD inhibitor D-APV in WT mice (Figure 4C) and in mice lacking the GluN2B CTD (Figure 4E) suggest that there is constitutive activity in the absence of synaptic stimulation. Consistent with this interpretation, we find that replacing the GluN2A CTD with the GluN2B CTD, which presumably augments this constitutive signaling, also reduces bulk protein synthesis to levels comparable to those observed in WT when stimulated with NMDA (Figure 4F). However, we did not perform the basal protein synthesis measurements in the presence of MK-801 in the A2B mice and therefore cannot rule out an additional contribution of ionotropic signaling to this decrease.^62,83,84^ It would be interesting to know whether additional stimulation with NMDA in the A2B mice would further suppress protein synthesis beyond the constitutive reduction.

Many studies have used selective agonists such as NMDA and DHPG to induce plasticity in the hippocampal slice. The advantage of this method is that it elicits changes at a large population of Schaffer collateral synapses, not just those activated by a stimulating electrode, and this enables coordinated biochemical, structural, and electrophysiological analysis of synaptic plasticity. Agonist-induced mGluR- and NMDAR-dependent LTD occludes and is occluded by LTD induced with electrical stimulation, indicating a shared expression mechanism.^4,47,85^ Although similar occlusion experiments have not been performed with structural plasticity, it has been shown that spines shrink in response to glutamate uncaging at individual synapses,^86^ and these changes are triggered by non-ionotropic activation of NMDARs.^87^ It therefore seems very likely that the conclusions of our study apply regardless of the route of induction, but this should be confirmed in future studies.

A strength of this study is the use of multiple approaches to confirm our conclusions. A case in point is the conclusion that activation of GluN2B is necessary and sufficient to account for the mNMDAR signaling leading to spine shrinkage. It is necessary because spine changes in response to NMDA are eliminated by genetic deletion of GluN2B (Figure 1E), pharmacological blockade of the ligand binding site on GluN2B (Figure 2C), and replacing the GluN2B CTD (Figure 3E). It is sufficient because there is no effect of genetic elimination of GluN2A (Figure 1A_ii_), pharmacological inhibition of GluN2A (Figure 2E_ii_), or replacing the GluN2A CTD (Figure 3B). Although it was not an explicit aim of this study, our experiments also shed light on the contributions of GluN2A and GluN2B subunits on functional LTD induced by NMDA. The finding that selective inhibition of ligand binding to GluN2B with conantokin-G^88^ eliminated LTD suggests that GluN2A di-heteromeric receptor activation is not sufficient to support induction (Figure 2C). Similarly, although residual LTD is observed in the *Grin2A* KO (Figure 1A), it does not appear that GluN2B di-heteromeric receptors are necessary, as LTD is unaffected by 30 μM Ro25-6891, a GluN2B-negative allosteric modulator that preferentially targets these receptors^39^ (Figure S1). Our data support the notion that the primary trigger for LTD is activation of GluN2A/B tri-heteromeric receptors, which have been shown to contribute 50% or more of the ion flux triggered by synaptic activation of NMDARs in CA1.^39,89^ These receptors are strongly inhibited by conantokin-G^88^ whereas ion flux through them is only partially inhibited by MPX-004 (30 μM),^36^ paralleling the effects of these compounds on LTD (Figures 2C and 2E). This conclusion is provisional, however, as further work is required to fully characterize the selectivity and potency of the compounds used in our study.

### RESOURCE AVAILABILITY

#### Lead contact

Requests for further information, resources, and reagents should be directed to and will be fulfilled by the lead contact, Mark Bear (mbear@mit.edu).

#### Materials availability

This study did not generate new unique reagents.

#### Data and code availability

All data reported in this paper will be shared by the lead contact upon request. This paper does not report original code. Any additional information required to reanalyze the data reported in this paper is available from the lead author upon request.

## STAR★METHODS

### EXPERIMENTAL MODEL AND STUDY PARTICIPANT DETAILS

All mice were generated and/or bred for at least 6 generations on a C57BL6/J (003025) background obtained from The Jackson Laboratory. Experimental cohorts for all mouse lines were P25-35 at the time of experiments, except AGS experiments where mice were P19-25. They consisted of female and male littermates, except *Fmr1* KO lines where only male mice were used. Homozygote Grin2Afl/fl and Grin2B fl/fl mice were maintained on a C57BL6/J background. To generate GluN2A KO, Grin2A(^2BCTD^) or Grin2B (^2ACTD^) Thy1-GFP littermate pairs, the stock line was initially crossed with Thy1-GFP mice. Heterozygous progeny, for Thy1-GFP and mutant gene of interest, were used to breed experimental mice to produce WT and KO littermates for experiments. To generate Grin2A(^2BCTD^) or Grin2B (^2ACTD^) mice, heterozygous males and females were crossed to produce WT, heterozygous and homozygous littermates, which were used for metabolic labeling experiments. Generation of double mutant lines involved crossing *Fmr1* heterozygous females with GluN2A^(2BCTD)^ heterozygous males, producing WT, Fmr1 KO, Grin2A(^2BCTD^) heterozygotes and Fmr1 KO/Grin2A(^2BCTD^) heterozygote double mutants, with only first-generation litters taken. All experiments were performed blind to genotype using age-matched littermate controls. At MIT, all animal care and procedures were approved by the MIT committee on Animal Care (CAC) and the Department of comparative medicine (DCM); they are also in compliance with the NIH “Guide for the care and use of laboratory animals”. At the University of Edinburgh all animal husbandry was carried out by University of Edinburgh technical staff. All animal procedures were performed in accordance the regulations set by the University of Edinburgh and the UK Animals Act 1986. At both institutes mice were group housed with access to unrestricted food and water and maintained on a 12 h light-dark cycle.

### METHOD DETAILS

#### CA1 viral infusions

Juvenile Grin2Afl/fl and Grin2B fl/fl mice (P25-30) were anesthetized and received subcutaneous injections of preoperative analgesics (buprenorphine (0.1 mg/kg, meloxicam; 1 mg/kg, and 1% lidocaine) before being head-fixed on a stereotaxic frame. Craniotomies were performed over CA1 where 20 short pulses of 20 nL of herpes simplex virus (HSV) expressing a cre-eGFP fusion protein (HSV-eGFP-cre) or HSV-eGFP as a control (~8.75x10^8^ particles/ml) was injected at a rate of 43 nL/min at 2–3 sites using a Nanoject III (Drummond Scientific) and a beveled glass injection pipette. After surgery mice received post-operative analgesics and were returned to their home cage. Around 5–7 days post injection, mice were sacrificed for recordings. Cre expression was generally limited to a sparse population of CA1 pyramidal cells in the dorsal hippocampus.

#### Preparation of hippocampal slices

Mice were anesthetized through isoflurane inhalation, sacrificed and hippocampi were isolated. At MIT, acute dorsal hippocampal slices (350 μm thick) were prepared using a vibratome (Leica Microsystems) in ice-cold dissection buffer containing (in mM): NaCl 87, sucrose 75, KCl 2.5, NaH_2_PO_4_ 1.25, NaHCO_3_ 25, CaCl_2_ 0.5, MgCl_2_ 7, ascorbic acid 1.3, and D-glucose 10, saturated with 95% O_2_, 5% CO_2_ (Sigma-Aldrich). For LTD and spine shrinkage experiments, the CA3 region was removed. Slices were initially recovered for 30 min at 32.5 °C then at room temperature for 2.5 h in artificial cerebrospinal fluid (ACSF) containing (in mM): NaCl 124, NaH_2_PO_4_ 1.2, KCl 5, NaHCO_3_ 26, glucose 10 CaCl_2_ 2, MgCl_2_ 1, saturated with 95% O_2_, 5% CO_2_. At the University of Edinburgh, horizontal hippocampal slices (400 μm thick) were prepared in ice-cold ACSF containing NaCl, 86; NaH_2_PO_4_, 1.2; KCl, 25; NaHCO_3_, 25; glucose, 20; sucrose, 75; CaCl_2_, 0.5; MgCl_2_, 7; saturated with 95% O_2_, 5% CO_2_ (Sigma-Aldrich). Slices were hemisected and an incision made through the CA1/CA3 boundary. Slices were initially recovered for 30 min at 32°C then at room temperature for 2.5 h in ACSF containing (in mM): NaCl, 124; NaH_2_PO_4_, 1.2; KCl, 2.5; NaHCO_3_, 25; glucose, 20; CaCl_2_, 2; MgCl_2_; 1, saturated with 95% O_2_, 5% CO_2_.

#### Electrophysiology

At MIT, field potential recordings were performed in a submersion chamber, perfused with ACSF (3–4 mL/min) at 30°C. fEPSPs were recorded in CA1 stratum radiatum with extracellular electrodes filled with ACSF. Baseline responses were evoked by stimulation of the Schaffer collaterals at 0.033 Hz with a two-contact cluster electrode (FHC, Bowdoin, ME) using a 0.2 m stimulus yielding 40–60% of the maximal response. Field recordings were filtered at 2 kHz, digitized at 50 kHz and analyzed using pClamp10 (Axon Instruments). Hippocampal slices were pre-incubated in ACSF containing either vehicle or GluN2 subtype specific blockers conantokin-G (2 μM), Ro25-6981 (30 μM) and MPX-004 (30 μM) for 40 mins prior to recording, and then continually perfused throughout the entire experiment. NMDAR-dependent LTD and spine shrinkage was induced by the acute application of NMDA (20 μM, 3 min). The initial slope of the response was used to assess changes in synaptic strength. Functional LTD was quantified by comparing the average response 50–60 mins after NMDA, to the average of the last 10 mins of baseline. Isolated NMDA-mediated fEPSPs were obtained in ACSF containing Mg^2+^ (0.3 mM) glycine (1 μM), picrotoxin (100 μM) and NBQX (20 μM) before perfusing conantokin-G (2 μM), Ro25-6981 (30 μM) or MPX-004 (30 μM).

For extracellular recordings in CA3, an ACSF filled electrode was placed in the stratum pyramidal layer of CA3. Responses were recorded using PClamp10 software in voltage-clamp, amplified x1000, filtered between 300 Hz and 10 kHz and digitized at 25 kHz. A 10-min baseline was recorded in ACSF before perfusing in either the GABA_A_ antagonist bicuculline (50 μM) or the group 1 mGluR_1/5_ agonist DHPG (50 μM). Voltage traces were Butterworth filtered between 300 and 1000 Hz before being downsampled by a factor of 10. The spike threshold was determined by calculating the median absolute deviation and multiplying by a factor of 5. Following spike detection, spikes were summed across 100 msec bins to reduce computational expense. To detect bursting events, a 500 msec sliding window was pulled across the entirety of the recording, from 5 to 60 min following the baseline period. A burst was identified if the spike rate within the window exceeded 8 Hz and was terminated in subsequent windows when the spike rate fell below 4 Hz.

At the University of Edinburgh, extracellular recordings in CA1 were performed by placing slices in a submersion chamber heated to 30°C (Fine Science Tools) and perfused with pre-oxygenated ACSF at a rate of 4 mL/min mGluR-dependent LTD was induced in CA1 by perfusing the slice with the group 1 mGluR agonist DHPG (50 μM, 5 min). Waveform data was collected using WinLTP 1999–2009 (WinLTP Ltd., University of Bristol), amplified 1000 times (npi electronics), filtered at 1.3 kHz and digitized (National Instruments) at 20 kHz. The data was exported to Microsoft Excel and the magnitude of LTD was calculated from the last 10 min of the recording relative to the pre-drug baseline.

#### Two-photon laser-scanning microscopy

Time-lapse fluorescence two-photon imaging was performed using a Prairie Technologies Ultima system attached to an Olympus BX-51WI that was equipped with a mode-lock femtosecond-pulse Ti:Sapphire laser (Chameleon, Coherent). Experiments have been previously described in detail in.^24^ Briefly, GFP was excited at 930 nm and images were taken with a 60X 0.9 NA objective lens, and a digital zoom of ×5.60 every 4 min.

#### Metabolic labeling

Hippocampi were rapidly dissected from mice and transverse hippocampal slices (500 μm) were prepared using a Stoelting tissue slicer in ice-cold artificial cerebrospinal fluid (ACSF) containing (in mM): NaCl, 124; NaH_2_PO_4_, 1.25; KCl, 3; NaHCO_3_, 26; glucose, 10; CaCl_2_, 2; MgCl_2_, 1, saturated in 95% O_2_ and 5% CO_2_. Hippocampal slices were left to recover for at least 4 h at 32°C in oxygenated ACSF. Transcription was blocked with actinomycin D (ActD, 25 μM; Tocris) ± drug for 30 min then slices were metabolically labeled in ^35^S-Methionine/Cysteine express protein labeling mix (PerkinElmer) ± drug for 30 min and snap frozen. Samples were homogenized in ice-cold buffer containing (in mM): HEPES, 10 pH 7.4; EDTA, 2; EGTA, 2; 1% Triton X-100 (Sigma-Aldrich); protease and phosphatase inhibitors. Proteins were precipitated with TCA (Sigma-Aldrich) for 10 min on ice before being centrifuged at 16,000 rpm for 10 min and resolubilised in 1 M NaOH. The pH was adjusted with 0.33 M HCl and each sample was added to scintillation cocktail in triplicate and counts per minute (CPM) were quantified and normalised to overall protein using a DC protein assay kit II (Biorad). Inter-experimental variation in incorporation rates were corrected by normalising each value to the ^35^S-Met/Cys ACSF and the average incorporation of all slices analyzed.

#### Image analysis

Throughout the baseline and LTD recording, a 512x512 pixel XY-scanned z series was taken every 4 min, at 1 μm of tissue depth for 15 μm of depth in total. Maximal fluorescence intensity of the z series was summed to obtain a z stack for every time point and collated to form a movie montage, aligned using the StackReg function in Fiji/ImageJ (by Rasband, W.S., U. S. National Institutes of Health). Typically, 15 spines on multiple dendritic regions were tracked throughout the recording by outlining a 20 x 20 pixel region of interest (ROI). Within this ROI, the total integrated fluorescence intensity of the green was calculated using ImageJ. Background fluorescence was tracked at 3 locations and subtracted, then overall fluorescence fluctuations was corrected by measuring the intensity over time at 4 locations. These values were then taken to be proportional to spine volume. As in our previous study,^24^ experiments showing >7% of drift variation during the baseline period, calculated by fitting a linear regression line for the 30 min of baseline were excluded from analysis.

#### Audiogenic seizures

Male Fmr1 KO and WT mice (P19-25) were weighed and injected intraperitoneally (i.p.) with 1 mg/kg Glyx-13 or vehicle (saline) 1 h prior to testing. Mice were habituated in the test chamber for 1 min before they were exposed to a 120 dB audiogenic stimulation (recorded sampling of a modified personal alarm) for 2 min, which was monitored with a decibel meter. All genotype and treatment conditions were tested within a session, with the experimenter blind to genotype and treatment. Incidence of seizures was scored based on whether the mice exhibited wild running, clonic seizure or tonic seizure.

### QUANTIFICATION AND STATISTICAL ANALYSIS

All data is expressed as a mean ± S.E.M with N representing the number of animals. Statistical analyses were performed using GraphPad Prism 9.0 with a confidence level set at 95%. Outliers (±2 SD from the mean) were removed and significant effects determined using Student’s *t*-test or one-way ANOVA with Bonferroni *post-hoc* test (single comparisons) or repeated-measures two-way ANOVA followed by paired Student’s *t*-test (multiple comparisons). AGS incidence scores were analyzed using Fisher’s Exact test.

## Supplementary Material

1

## Figures and Tables

**Table:** KEY RESOURCES TABLE

REAGENT or RESOURCE	SOURCE	IDENTIFIER
Bacterial and virus strains		
ST HSV-cre-eGFP	Rachael Neve, Massachusetts General Hospital Gene Delivery Technology Core	RN5
ST HSV-eGFP	Rachael Neve, Massachusetts General Hospital Gene Delivery Technology Core	RN1
Chemicals, peptides, and recombinant proteins		
NMDA	Sigma-Aldrich	M3262
Picrotoxin	Sigma-Aldrich	P1675
NBQX	Sigma-Aldrich	N183/1044
Glycine	Sigma-Aldrich	G7126
Conantokin-G	Tocris	4136
MPX-004	Alomone	M-280
Ro25-6981	Tocris	1594
D-APV	Tocris	0106
Bicuculline	Sigma	14343
Actinomycin D	Tocris	1229
DHPG (U.of.E)	Sigma-Aldrich	D3689
DHPG (M.I.T)	Tocris	0805
MK-801	Sigma-Aldrich	M107
Glyx-13	Tocris	3406
Critical commercial assays		
DC Protein Assay kit II	Bio-Rad	5000112
EasyTag EXPRESS 35S Protein Labeling Kit	PerkinElmer	NEG772014MC
Experimental models: Organisms/strains		
Mouse: Thy1-GFP	The Jackson Laboratory Feng et al.^90^	011070; RRID:IMSR_JAX:011070
Mouse: Grin2Afl/fl Grin2Bfl/fl	Gray et al.^33^	N/A
Mouse: GluN2A KO	Townsend et al.^91^	N/A
Mouse: Grin2A(2BCTD) Grin2B(2ACTD)	Ryan et al.^40^	N/A
Mouse: Fmr1 KO	The Jackson Laboratory	003025; RRID:IMSR_JAX:003025
Mouse: C57BL6/J	The Jackson Laboratory	003025
Software and algorithms		
Graphpad Prism 9.0	GraphPad	N/A
ImageJ	Schneider et al.	https://imagej.nih.gov/ij/
PClamp10	Axon Instruments	N/A
MATLAB R2023a	MathWorks	N/A
Microsoft Excel	Microsoft Office 2011	N/A
WinLTP 1999-2009	WinLTP Ltd., University of Bristol	N/A

## Highlights

- Non-ionotropic NMDAR signaling regulates protein synthesis and dendritic spine morphology
- This form of signaling is mediated by the GluN2B carboxy-terminal domain
- Eliminating GluN2B signaling phenocopies aspects of fragile X syndrome
- Augmentation of GluN2B signaling reverses core phenotypes in the *Fmr1* KO mouse
