# Supplementary material for: Non-ionotropic signaling through the NMDA receptor GluN2B carboxy-terminal domain drives dendritic spine plasticity and reverses fragile X phenotypes
Source: Cell Rep. Author manuscript; Available in PMC 2025 Apr 18. (PMC12006837; doi:10.1016/j.celrep.2025.115311)
Supplement: 1 [file NIHMS2069103-supplement-1.pdf]

Cell Reports, Volume *44*

Supplemental information

Non-ionotropic signaling through the NMDA receptor GluN2B carboxy-terminal domain drives dendritic spine plasticity and reverses fragile X phenotypes

Stephanie A. Barnes, Aurore Thomazeau, Peter S.B. Finnie, Maxwell J. Heinrich, Arnold J. Heynen, Noburu H. Komiyama, Seth G.N. Grant, Frank S. Menniti, Emily K. Osterweil, and Mark F. Bear

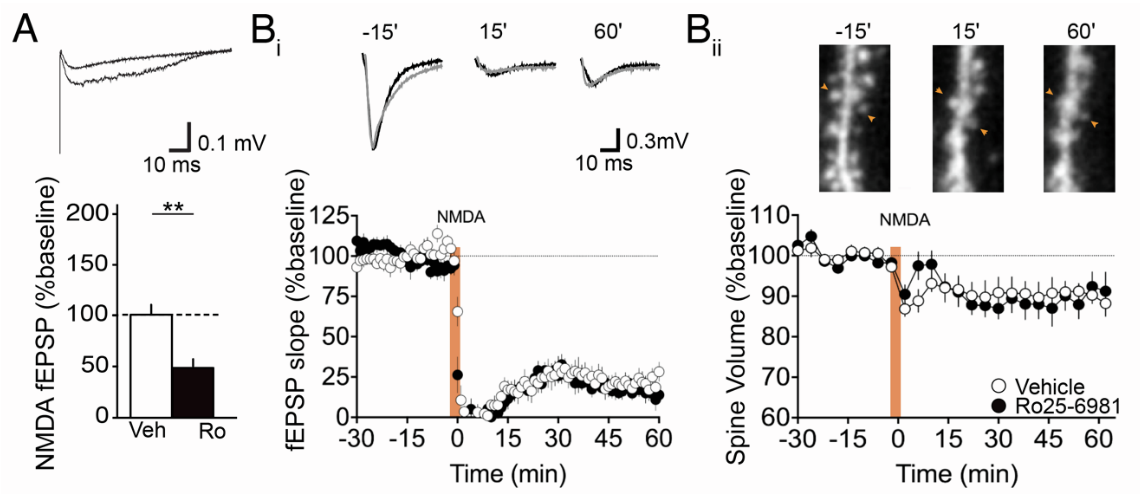

Supplemental Figure 1: NMDA-induced LTD and spine shrinkage is unaffected by negative allosteric modulation of the GluN2B N-terminal domain. The negative allosteric modulator Ro25-6981, targets the N-terminal domain of GluN2B containing NMDARs, limiting ion flux through the receptor. (**A**) Ro25-6981 (30 μM) eliminated 51% of the isolated NMDAR-mediated fEPSP (WT vehicle: 100 ± 10%; WT Ro25-6981: 49 ± 7%, n = 8; p = 0.0010, unpaired t-test) but had no effect on either LTD (**Bi**; WT vehicle: 21 ± 5%, n = 8; WT Ro25-6981: 15 ± 3%, n = 8; p = 0.225, unpaired t-test) or spine shrinkage (**Bii**; WT vehicle: 90 ± 3%, n = 8; WT Ro25-6981: 89 ± 4%, n = 8; p = 0.95, unpaired t-test). Representative fEPSP traces and images are shown 15 mins before, 15 mins and 60 mins after LTD induction.

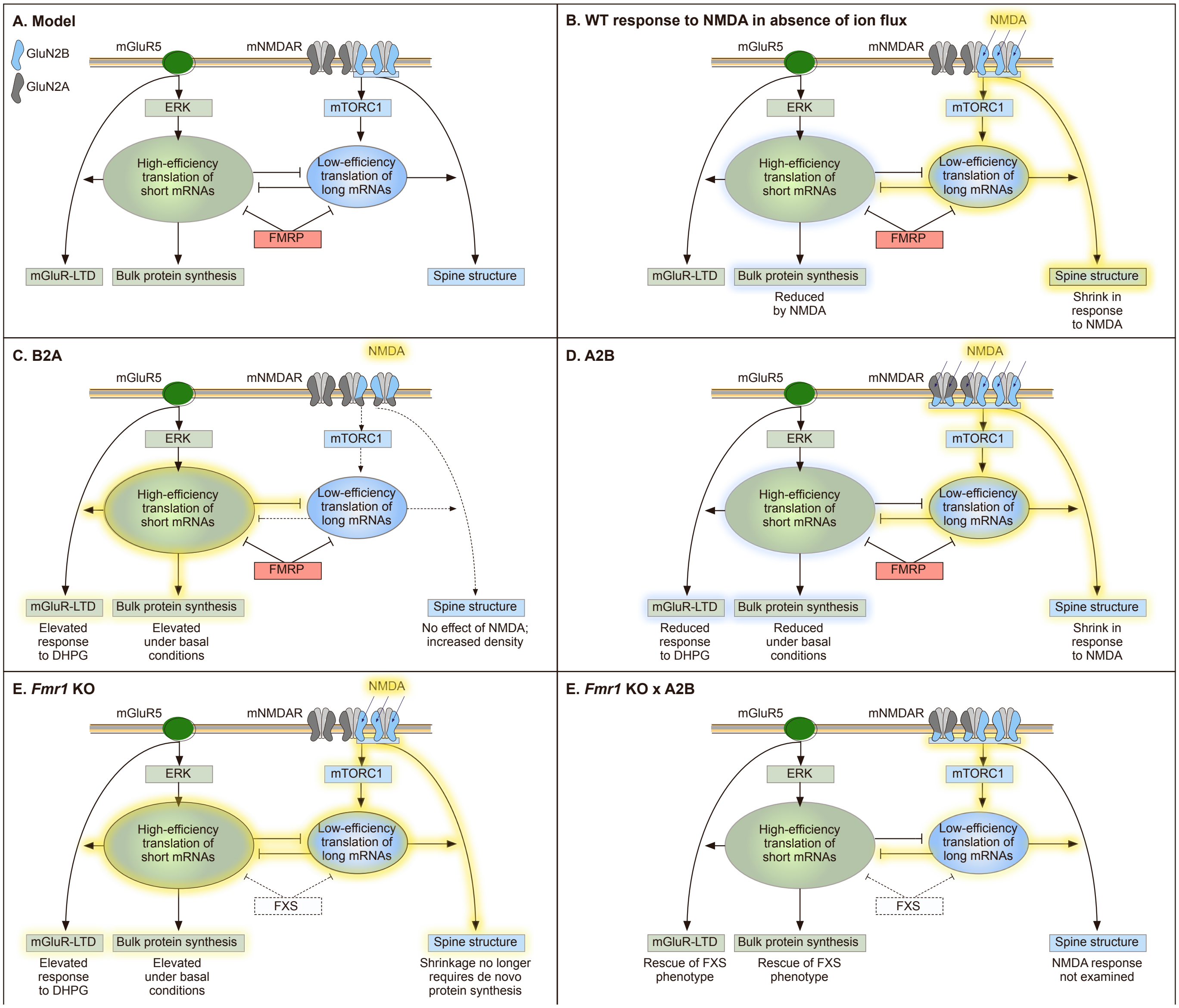

Supplemental figure 2 Model and summary
